# Agency in Family Planning: A Scoping Review of the Measurement of Agency in Low‐ and Middle‐Income Countries

**DOI:** 10.1111/sifp.70025

**Published:** 2025-07-09

**Authors:** Francine Wood, Courtney McLarnon, Sarah Smith, Nitya Yerabandi, Lotus McDougal

## Abstract

Improving women's agency in family planning is an integral component of empowerment, requiring culturally relevant, reliable, and valid measures. Measuring agency—action towards the achievement of self‐determined goals—is key to tracking progress as highlighted by its inclusion in the Sustainable Development Goals. Yet, agency measurement within low‐ and middle‐income contexts has all too often involved applying measures developed and tested in high‐income contexts, and conceptual confusion has also led to both overlapping measures and those that omit key facets of agency. To examine the construction and application of agency measures in family planning research and programs, we conducted a scoping review of studies in low‐and middle‐income countries. Of 9,289 articles and abstracts screened, 72 met our inclusion criteria and included family planning outcomes. We identified 58 unique measures. Most measures were summative and described psychometric testing. Measures often included family planning‐specific items, generally focused on contraceptive use with less attention to areas such as communication, access to services, or fertility timing. While increased interest in measuring family planning agency is evident, inconsistencies in measurement hinder cross‐contextual comparisons. As family planning research and programs adopt empowerment‐focused benchmarks, validated measures of agency are needed to accurately assess impact.

## INTRODUCTION

In the more than 30 years since the International Conference on Population and Development, agency and empowerment have become increasingly recognized as central to sustainable development, health, and well‐being (Chandra‐Mouli et al. 2015; United Nations 1995, 2015). This is particularly important in family planning, which has historically been beset by inequities based on gendered power dynamics and inequitable social and gender norms (Gupta et al. 2019; Taukobong et al. 2016; Hay et al. 2019). The centering of agency within family planning discourse is an important turning point in shifting dialogues which have historically focused on sometimes coercive and incentivized means of population control to a rights‐based approach that prioritizes person‐centered programming within an enabling environment (Ojong et al. 2024; The Lancet 2019; Brown and Hardee 2024).

Agency is situated within the heart of the empowerment process, operating within individual, relational, and collective realms (Raj et al. 2024). Recent research guided by multidisciplinary scholarship and input broadly defines agency as encompassing the efficacy of individuals or groups to engage in action (can), the ability to enact actions aligned with their choices or goals (act), and the resilience to continue these actions in the face of external opposition (resist) (Raj et al. 2024). While many agency frameworks and conceptualizations have been proposed, there is a general consensus that agency has multiple facets and is influenced by various interconnected factors, including an individual's motivations, beliefs, and values (Edmeades et al. 2018; National Academies of Sciences Engineering and Medicine 2025; Yount et al. 2020; Kabeer 1999; Donald et al. 2020). Agency is often preceded by critical consciousness, or the awareness that there is a choice or decision available (Freire 2020), and the process of setting an individual or collective goal (Ryan and Deci 2006). Furthermore, individual and collective agency exists in complex and dynamic contexts and as such, are influenced by societal and cultural norms (Hay et al. 2019) as well as internal and external resources such as affect, resilience, and social support (Raj et al. 2024). Though agency is often discussed at the individual level, it can equally exist within couples, communities, and broader collectives (Donald et al. 2020).

Agency is thus a cornerstone of family planning, embodying the ability of individuals to make informed decisions about their reproductive lives and to set and work towards their self‐determined reproductive goals (Family Planning 2020 Rights & Empowerment Working Group 2014; Bhan and Raj 2021). At its essence, agency empowers women to navigate a complex family planning landscape, including decisions about whether, when, and what type of contraception to use; fertility; and reproductive health services. Agency is the means through which people can exercise choice and action to achieve their reproductive goals; the freedom to work towards these goals, and to set the goals they desire in their unique circumstances, is an important component of enabling women to assert their reproductive rights and make decisions that align with their individual circumstances (Holt et al. 2024; Raj et al. 2024).

While progress has been made in recent years, gaps in our understanding and measurement of agency remain (Bhan et al. 2022). A recent review identified theoretical and measurement challenges related to agency and norms in family planning, including the lack of conceptual clarity on what agency means to people across diverse settings, as well as the application of agency measures developed in high‐income contexts within low‐and middle‐income contexts (Bhan et al. 2020). Many existing measures of agency capture only some aspects, impeding our understanding of the complex nature of agency around family planning needs and choices of men and women in different settings around the world. For instance, several measures focus on an individual's involvement in decision‐making but fail to understand other key aspects such as their family planning goals, self‐efficacy to achieve those goals, experiences of opposition when acting on their family planning goals, or their level of satisfaction with or preference for being involved in decisions or having others involved in decisions (Holt et al. 2024; Raj et al. 2024; Hinson et al. 2019). With the increased interest in understanding agency around family planning and more broadly reproductive health, uncovering these elements of agency allows measures to capture more of that complexity and be more adept at informing family planning programs, messaging, and research.

These gaps in the measurement of agency are not limited to family planning; at present, there are no reviews or protocols that focus specifically on the measurement of men's, women's, or children's agency in development contexts across domains of health (Asghar, McLarnon, and McDougal 2023). To address these deficits, the USAID‐funded Agency for All Project conducted a scoping review of published literature to review and assess current conceptualizations of agency and identify measures of agency being used in low‐ and middle‐income settings across multiple domains of health, including family planning. This review was conducted to inform subsequent agency measure development activities in West Africa, East Africa, and South Asia. The findings of this larger review across multiple health domains are being analyzed and published separately. This paper focuses exclusively on scoping review findings related to the measurement of agency in global family planning research and programming within low‐ and middle‐income countries.

## METHODS

The guiding research question for this analysis was: How has agency been quantitatively measured to date in different domains of global health and well‐being? Specifically, this paper examines the quantitative measurement of agency in global family planning research and programming. To answer this, we analyzed family planning literature identified during the extraction process, which is detailed in the sections below, along with our overall search process. We operationalize our definition of family planning following Fabic (2022), namely “The services, policies, information, attitudes, practices, and commodities, including contraceptives, that give individuals who desire to avoid pregnancy the ability to do so.”

This scoping review was conducted in accordance with Joanna Briggs Institute scoping review methodologies (Peters et al. 2020), and the review protocol has been registered with Open Science Framework (Asghar, McLarnon, and McDougal 2023). We followed reporting guidelines as described in the PRISMA extension for scoping reviews (PRISMA‐ScR) to ensure the methodological and reporting quality of our findings (Tricco et al. 2018).

### Data Sources and Search Strategy

We used a systematic approach that included a comprehensive search for peer‐reviewed articles in four electronic bibliographic databases (PubMed, CINAHL, PsycINFO, and ProQuest), and a manual search of the Evidence‐based Measures of Empowerment for Research on Gender and Equality (EMERGE) database. Our search strategy was focused on five keywords—agency, empowerment, measurement, conceptualization, health—and related search terms such as personal autonomy, participant participation, self‐efficacy, decision making, negotiation, collective power, power to, dimensional measurement accuracy, conceptual understanding, validity, maternal and child health, adolescent health, reproductive health, and nutrition. We used Boolean operators (AND/OR) and truncations (“”, *) depending on the database (see Tables T1–T4 in the Online Appendix for the search terms). Preprint articles included during the search were updated with publication dates and citation information as papers were published during the extraction process in 2023. In addition to searching electronic databases and the EMERGE database, we consulted with expert researchers and implementors on agency and empowerment to identify relevant gray literature.

### Eligibility Criteria

Articles were eligible for inclusion if they were written in English, published between January 1, 2000, and November 30, 2022, focused on populations between 14 and 65 years of age residing in low‐ and middle‐income countries, and included a focus on one or more of the following health domains: reproductive, maternal, newborn and child health, adolescent health, mental health, nutrition, sexual health and family planning, HIV/AIDS, and interpersonal or community violence. Additionally, articles had to either explicitly include agency measures (including survey items and response options) in the article text or include discussions of agency conceptualizations. Any article or publication that did not meet all inclusion criteria was excluded.

### Study Selection

All titles and abstracts were uploaded to Covidence systematic review software (Veritas Health Innovation 2023) and reviewed for duplicates. A team of 13 reviewers individually screened titles and abstracts of the identified publications based on the study criteria. Following title/abstract screening, seven reviewers conducted full article eligibility screening. At the full‐text review stage, two reviewers independently screened articles; conflicts that arose were resolved either by the senior researchers or through team consensus, following a decision‐making flow chart.

Prior to the screening stage, the review team participated in training sessions to review the protocol, processes involved in each stage, definitions of the types of agency and related constructs, as well as the health outcomes of interest. Agency constructs were defined in accordance with the recent multidisciplinary research (Raj et al. 2024), and examples of each construct were provided for each of these constructs in the protocol, Covidence, and several scoping review documents. For instance, examples of the “can” construct included references to self‐efficacy, self‐esteem, or confidence, while the “act” construct included references to decision‐making or action to pursue goals, and the “resist” construct included references to resistance to opposition. Additionally, the team held regular meetings to ensure a shared understanding of terms and address any issues that arose during the various stages.

### Data Extraction

Data were extracted from articles meeting inclusion criteria using an extraction template in Covidence and was completed by a team of four reviewers. Extracted data from each article included the abstract; author(s); title; year of publication; citation; study context; study design (i.e., study type (qualitative, quantitative, mixed methods, etc.); data source and study population age and gender); study health domain (e.g., sexual and reproductive health, family planning, maternal health, etc.); types of agency measured in terms of construct (can, act, resist), level of social ecology (individual, community, organizational), and related constructs (e.g., critical consciousness, aspiration and goal‐setting, social norms); health domain; agency conceptualizations; agency measures (items and responses); measure adaptation; measure construction (categorical or summative) and whether or not measure psychometrics (e.g., reliability, validity) were assessed. When applicable, we noted if the study was conducted among historically marginalized and oppressed populations such as sex workers, low‐income or affected by poverty, and individuals affected by conflict. An individual team member extracted information from the articles and was reviewed for completeness and accuracy by the study leads.

### Data Summary and Synthesis

The extracted data were transferred from Covidence and into an Excel spreadsheet, which then underwent a cleaning process to identify missing information and ensure that no duplicates were included. As this scoping review extracted information on both qualitative conceptualizations of agency and quantitative measures of agency, the two components were separated at this stage of analysis. Qualitative conceptualizations of agency were analyzed separately and are outside the scope of the present analysis.

The analysis presented in this paper was conducted to respond to the guiding research question as to how agency has been quantitatively measured to date across global health domains. Accordingly, after the cleaning process, we compiled the extracted family planning literature into a separate Excel spreadsheet and information extracted from these articles formed the basis of our analysis. To analyze the data, descriptive statistics were calculated to summarize the characteristics of the studies and agency constructs within these studies. Unique measures within the studies were organized based on whether they were agency constructs, related constructs, or a combination of both. Agency constructs are those within the “can,” “act,” and “resist,” framework, while related constructs are constructs proximally related to agency in the empowerment process including critical consciousness, aspiration, social norms, conviction, autonomy, and social support. Classifications as agency constructs or related constructs were completed during data extraction and were based on the individual measure items. A given measure could be coded under multiple categories. Additionally, further review of the measure items was conducted to explore the health domains within which agency and related constructs were measured.

## RESULTS

After the search of articles, 10,129 were identified as potentially relevant records and 903 were removed after deduplication. More than half of these articles were found to be irrelevant as they did not meet our inclusion criteria and were excluded during the title and abstract screening stage (*n* = 6,530); the remaining 2,696 were deemed full‐text eligible. Many articles were excluded during the full‐text review process because they did not meet the inclusion criteria. For example, 195 studies were excluded because they were conducted in high‐income countries, 758 articles did not include our specific health domains of interest, 686 quantitative studies did not include the survey items and response options for the agency measures, 69 qualitative articles did not include conceptualizations of agency, and 16 were not in English. We also excluded articles if we could not access the entire paper (*n* = 103) or could not access the scale items (*n* = 90).

**FIGURE 1:**
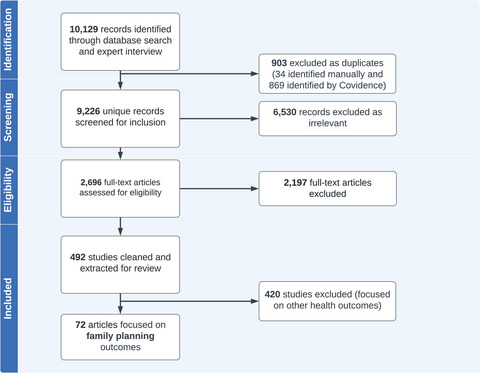
PRISMA flowchart of study selection and extraction

After our thorough and systematic process, we deemed 492 articles eligible for extraction. Of these, 424 included quantitative measures of agency. During extraction, we identified health domains, including family planning, discussed within each publication. This analysis presents findings from the 72 articles that included measures of agency focused within the family planning health domain. Figure 1 shows a PRISMA flow chart showing our article selection process from identification to final inclusion in this paper.

### General Characteristics of Included Articles

While all articles within the family planning domain (*n* = 72) were published between 2000 and 2023, nearly two‐thirds (65 percent) were more recent, published after 2015 (Table 1). The majority of studies explored family planning outcomes in tandem with other health outcomes (86 percent) such as sexual and reproductive health, nutrition, and intimate partner violence. Studies most often explored family planning along with reproductive health domains (69 percent), social relationships (42 percent), and sexual health (24 percent). Most studies used a quantitative study design (88 percent) and primary data sources (63 percent). Target populations were primarily women/girls (81 percent) or both women/girls and men/boys (17 percent). While over three‐fifths of the articles were conducted in sub‐Saharan Africa (65 percent), less than one‐tenth were conducted in East Asia and the Pacific (7 percent) and Europe and Central Asia (6 percent).

**TABLE 1:** General characteristics of articles included in the review (n=72)

Characteristic	*N*	%
Age groups^a^		
10–17 years	44	61.1
18–29 years	71	98.6
30–49 years	60	83.3
50–64 years	11	15.3
65+ years	2	2.8
Target population		
Women/girls	58	80.6
Men/boys	3	4.2
Women/girls and men/boys	12	16.7
Region^a^		
Sub‐Saharan Africa	47	65.3
East Asia and Pacific	5	6.9
Europe and Central Asia	4	5.6
Latin America and the Caribbean	8	11.1
Middle East and North Africa	12	16.7
South Asia	20	27.8
Context^a^		
Rural	44	61.1
Urban	44	61.1
Peri‐urban	16	22.2
Not specified	20	27.8
Study design		
Quantitative	63	87.5
Mixed methods	7	9.7
Other	2	2.8
Data source		
Primary research	45	62.5
Secondary analysis	27	37.5
Publication year		
2000–2005	6	8.3
2006–2010	5	6.9
2011–2015	13	18.1
2016–2020	31	43.1
2021–2023	17	23.6
Focal areas		
Family planning only	10	13.9
Family planning + other study domain	62	86.1
Study health domains^a^		
Sexual and reproductive health		
Reproductive health	50	69.4
Family planning	72	100.0
Maternal health	14	19.4
Neonatal health	2	2.8
Child health	6	8.3
Adolescent health	2	2.8
Sexual health	17	23.6
Social relationships	30	41.7
Intimate partner violence	5	6.9
Infectious diseases (HIV/AIDS)	1	1.4
Respectful care	1	1.4
General well‐being (mental health, nutrition, other)	7	2.8

^a^Categories listed are not mutually exclusive.

As shown in Table 2, the majority of studies discussed agency at the individual level (90 percent), while one in three discussed interpersonal agency (an individual's ability to achieve their goals within a relationship or in interaction with others—33 percent) and one in 10 discussed community agency (the ability of a group of people to act together towards a shared goal—11 percent). More than two‐thirds of studies included a focus on some sort of agentive action (“act”; 68 percent), while one‐third included measures of efficacy (“can,” 39 percent). Two of the 72 studies included measures encompassing reaction to backlash against agentive action (“resist,” 3 percent). In addition to the main agency constructs, several studies included related constructs of agency. One in five studies included a focus on social norms (21 percent), and few studies explored critical consciousness (1 percent), aspiration or goal setting (4 percent), or conviction (1 percent).

**TABLE 2:** Agency‐related characteristics of articles included in the review (*N* = 72)

	*n*	%
Agency constructs		
Can	28	38.9
Act	49	68.1
Resist	2	2.8
Related constructs		
Critical consciousness	1	1.4
Aspiration or goal‐setting	3	4.2
Conviction	1	1.4
Social norms	15	20.8
Other: ability, autonomy (financial, communication, and sexual),	7	9.7
Freedom of movement, motivation, social support		
Level of social ecology		
Individual	65	90.3
Interpersonal (relational/marital)	24	33.3
Collective	8	11.1

NOTE: Categories listed are not mutually exclusive.

### Agency Measures and Related Constructs Identified in Included Articles

Within the included 72 family planning‐focused articles that included measures of agency or related constructs, we identified 58 distinct measures of which 40 measured at least one agency construct and 28 measured at least one related construct.

There were 23 unique measures focusing on agentive action, 23 on efficacy, and two on backlash to agentive action. Agentive action measures predominantly centered on decision‐making power around contraceptive use and “resist” construct measures assessed the use of contraceptives despite external opposition. While measures related to the “can” construct primarily aimed at assessing self‐efficacy to use contraceptives, discuss contraceptive use, or negotiate sexual relations, a limited number explored self‐esteem and control over action. A list of all unique measures and corresponding characteristics identified in this review is presented in Table T5 in the Online Appendix.

**FIGURE 2:**
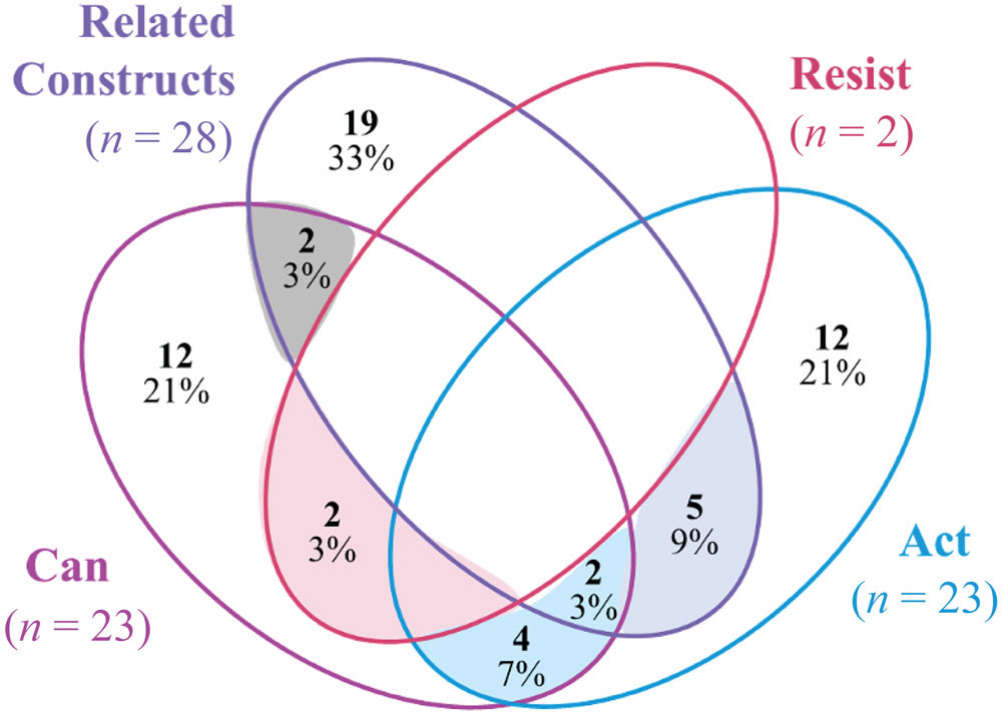
Venn diagram of measures of agency and related constructs within the unique measures (*n* = 58)

While the unique measures more commonly included only one agency construct or related constructs, there were several instances where unique measures frequently comprised multiple constructs of agency and related constructs, as illustrated in the shadings in Figure 2. Where there were multiple constructs included, the measure most commonly included two constructs of agency or an agency construct and a related construct. For example, the Family Planning Tool by Alemayehu et al. (2020) and the Pregnancy‐Related Empowerment Scale (Somji et al. 2022) included efficacy and agentive action, while the Reproductive Empowerment Scale (Mandal and Albert 2020) included efficacy, agentive action, and related constructs.

Unique agency measures were universally used among 18–29 year olds and also featured prominently in the 30–49 age group (Table 3). Women and girls were the primary focus of studies using these measures, and, of note, both measures of backlash to agentive action were used among women and girls. Across all three agency constructs, measures were most often used in sub‐Saharan Africa. “Can” measures were least commonly used in Europe, Pacific and East, Central and South Asia, while agentive action measures were least commonly used in Europe, East and Central Asia, Latin America, and the Caribbean. Measures focused on backlash to agentive action were only used in sub‐Saharan Africa, Latin America, and the Caribbean. As shown in Table T7 in the Online Appendix, measures used in sub‐Saharan Africa focused primarily on “can” followed closely by agentive action, while resistance to backlash was used once. In South Asia, agentive action was the most commonly used measure and none addressed resistance to backlash. In Latin America and the Caribbean, three‐fifths of the measures focused on “can,” two‐fifths on “act,” and one‐fifth on “resist,” whereas, in the Middle East and North Africa, an equal proportion of measures were focused on “can” and agentive action.

**TABLE 3:** Characteristics of unique measures of agency

	Can		Act		Resist		Total	
	*n*	%	*n*	%	*n*	%	*n*	%
Age groups^a^								
10–17 years	11	47.8	19	82.6	1	50.0	25	62.5
18–29 years	23	100.0	23	100.0	2	100.0	40	100.0
30–49 years	17	73.9	18	78.3	1	50.0	29	72.5
50–64 years	5	21.7	3	13.0	0	0.0	7	17.5
65+ years	1	4.3	1	4.3	0	0.0	1	2.5
Target population								
Women/girls	13	56.5	17	73.9	2	100.0	25	62.5
Men/boys	2	8.7	1	4.3	0	0.0	3	7.5
Women/girls and men/boys	8	34.8	5	21.7	0	0.0	12	30.0
Region^a^								
Sub‐Saharan Africa	18	78.3	14	60.9	1	50.0	26	65.0
East Asia and Pacific	2	8.7	1	4.3	0	0.0	3	7.5
Europe and Central Asia	1	4.3	2	8.7	0	0.0	3	7.5
Latin America and the Caribbean	3	13.0	2	8.7	1	50.0	5	12.5
Middle East and North Africa	3	13.0	3	13.0	0	0.0	6	15.0
South Asia	2	8.7	9	39.1	0	0.0	10	25.0
Context^a^								
Rural	10	43.5	18	78.3	1	50.0	24	60.0
Urban	9	39.1	15	65.2	1	50.0	20	50.0
Peri‐urban	7	30.4	5	21.7	0	0.0	9	22.5
Marginalized population	4	17.4	2	8.7	0	0.0	6	15.0
*N*	23	100.0	23	100.0	2	100.0	40	100.0

^a^ Categories listed are not mutually exclusive.

The use of measures in marginalized populations remains low, with documentation of only two unique agentive action measures—Transactional Sexual Relations by Fielding Miller (Fielding‐Miller et al. 2017) and Human Development Survey Decision Making Measure (Samanta 2020)—and four unique efficacy measures—Condom Use Self‐Efficacy Scale by Wagner (Wagner et al. 2014), Contraceptive Self‐Efficacy Scale by McCarthy (McCarthy et al. 2019), General Self‐Efficacy Scale by Wagner (Wagner et al. 2014), and the Work Self‐Efficacy Scale (Wagner et al. 2014)—being used among the marginalized population.

Unique measures on related constructs were focused on social norms (*n* = 14), autonomy (*n* = 6), aspiration or goal setting (*n* = 4), social support (*n* = 3), conviction (*n* = 2), and critical consciousness (*n* = 1). As shown in Table 4, more than half of the unique measures on related constructs were used among 18–29 and 30–49 year olds, women and girls, and in sub‐Saharan Africa. Related constructs were least predominant among men and boys and marginalized populations and were least often used in Central and East Asia, Europe, and the Pacific.

**TABLE 4:** Characteristics of unique measures of related constructs (*N* = 28)

	*n*	%
Age groups^a^		
10–17 years	13	46.4
18–29 years	27	96.4
30–49 years	18	64.3
50–64 years	2	7.1
65+ years	1	3.6
Target population		
Women/girls	20	71.4
Men/boys	1	3.6
Women/girls and men/boys	7	25.0
Region^a^		
Sub‐Saharan Africa	20	71.4
East Asia and Pacific	1	3.6
Europe and Central Asia	0	0.0
Latin America and the Caribbean	4	14.3
Middle East and North Africa	7	25.0
South Asia	8	28.6
Context^a^		
Rural	15	53.6
Urban	16	57.1
Peri‐urban	12	42.9
Marginalized population	2	7.1
*N*	28	100.0

^a^ Categories listed are not mutually exclusive.

As shown in Table 5, about a third of the unique agency measures were classified as original measures, having been developed specifically for the study and its context. Another third were not adapted and less than one‐fifth were adapted for the study context or population. The majority of the unique agency measures were developed as summative scores or scales. Among these summative measures, there was variability in the psychometric assessment of the scores. About half of the unique “can” construct measures (56 percent) were assessed for both reliability and validity, while 19 percent were assessed for reliability only and none were assessed for validity only. Two‐thirds of the unique agentive action measures were psychometrically evaluated, with about two‐fifths undergoing assessments for only reliability (19 percent), one‐tenth of assessments for only validity (10 percent), and half assessments for both reliability and validity (52 percent). Both unique measures focusing on backlash to agentive action were summative, with only one of them assessed solely for reliability. Notably, while many summative measures received some psychometric evaluation, a substantial portion of the unique summative measures lacked any form of assessment across all the three constructs of agency. About a third of the summative measures did not include assessments of reliability or validity (33 percent).

**TABLE 5:** Measure construction and psychometric testing of unique agency measures

	Can		Act		Resist		Total	
	*n*	%	*n*	%	*n*	%	*n*	%
Measure type								
Original	13	56.5	13	56.5	2	100.0	24	60.0
Adapted	5	21.7	4	17.4	0	0.0	7	17.5
Not adapted	5	21.7	6	26.1	0	0.0	10	25.0
Measure construction								
Binary or categorical	7	30.4	3	13.0	0	0.0	8	20.0
Summative	16	69.6	21	91.3	2	100.0	33	82.5
Psychometric properties (if summative)^a^								
None	4	25.0	7	33.3	1	50.0	11	33.3
Reliability only	4	18.8	4	19.0	1	50.0	8	24.2
Validity only	0	0.0	2	9.5	0	0.0	2	6.1
Reliability and validity	9	56.3	11	52.4	0	0.0	16	48.5
*N*	23	100.0	23	100.0	2	100.0	40	100.0

NOTE: Categories of agency (can, act, and resist) are not mutually exclusive.

^a^ Psychometric properties were assessed only if measures were summative.

Among the summative measures, two measures—Decision Making measures from the Demographic and Health Survey and the Contraceptive Self‐Efficacy Scale by Levinson, Sadigursky, and Erchak (2004)—were used multiple times with varying psychometric testing approaches each time. The Contraceptive Self‐Efficacy Scale by Levinson et al. with a focus on efficacy was solely assessed for reliability once and both reliability and validity twice. Demographic and Health Survey measures focused on agentive action were constructed 18 times as summative scores. Of these, four instances were psychometrically evaluated solely for reliability, five instances solely for validity, five instances for both validity and reliability, and in five instances no testing was conducted. The full list of Demographic and Health Survey measures and their psychometric properties are included in Table T6 in the Online Appendix.

Most of the unique measures included items tailored to family planning or reproductive health, with 44 of the 58 unique measures including items focused on these health areas (see Table T5 in the Online Appendix). Overall, the agency measures focusing on family planning outcomes centered around contraceptive use (Table 6), with a greater focus on measuring efficacy and agentive action to using contraception. Many of these measures, such as the Family Planning Tool (Alemayehu et al. 2020), the Reproductive Autonomy Scale (Loll et al. 2019), and the Women's and Girl's Empowerment–SRH Index (Moreau et al. 2020), have been developed to exclusively or partly measure contraceptive use. The only two measures that captured reaction to backlash against agentive action—the Family Planning Use and Discussion Self‐Efficacy Scale (Richardson 2014) and the Family Planning Self‐Efficacy Scale by Okigbo (Okigbo et al. 2018)—did so within the context of contraceptive use. While most measures assessed general contraceptive use, two measures focused on specific methods. The Partner IUD Use and Discussion Self‐Efficacy measure (Ha, Jayasuriya, and Owen 2003) aimed to measure discussion of IUD with a partner, and the Contraceptive Self‐Efficacy Scale (McCarthy et al. 2019) inquired about women's efficacy in using the pill, IUD, injection or implant. Together with contraceptive use, measures also focused on assessing individual's engagement in family planning discussions with partners, and in some cases, other family members (Wegs et al. 2016). Additionally, measures focused on assessing an individual's engagement in conversations about accessibility of family planning services, with a focus on ability to purchase contraceptives, and access a health facility or pharmacy to obtain a method and cost of contraception. For instance, the Family Planning Self‐Efficacy Scale by Okigbo et al. (2018) measured accessibility by asking for an individual's efficacy to obtain a method if they wanted to and they had access to a place where methods are offered, while the Contraceptive Self‐Efficacy Measure by Kahsay et al. (2018) asks if the cost of modern contraceptive is easy to overcome. Among agency‐related constructs, measures related to social norms around family planning, in particular, focused on assessing descriptive norms (perceptions of what people do) and injunctive norms (perceptions of approval by others) around contraceptive use were most common.

**TABLE 6:** Family planning and reproductive health domains in agency measures

	Contraceptive					Fertility		
	Use	Discussion	Access	Condom use	Sexual relations	Timing	Preferences	Other
Constructs								
Can	17	7	6	3	5	2	1	5
Act	9	2	1	0	6	5	2	10
Resist	2	0	0	0	0	0	0	0
Related constructs								
Aspiration or goal‐setting	4	1	0	1	2	1	1	0
Social norms	10	2	0	1	7	1	3	2
Conviction	1	1	0	0	0	0	0	0
Critical consciousness	1	1	0	0	1	0	0	0
Social support	2	1	0	0	1	0	0	1
Autonomy	3	1	0	0	2	2	1	2

NOTE: Health domains are not mutually exclusive

Sexual relations emerged as another key measurement domain, both for agentive action and efficacy, although it was more often reported as an agentive action. Studies were focused on measuring an individual's ability to decide on and discuss sexual relations with partners and peers. For instance, the Demographic and Health Survey asks women about their ability to refuse sex with their partner, the Family Planning and Gender Transformative Decision‐making scale (Wegs et al. 2016) includes items on decision‐making around the timing of sexual relations with a husband, and the Safer Sex Self‐Efficacy Scale (Mandal, Muralidharan, and Pappa 2017) includes items about talking about sex with a romantic partner or friend.

Another focal area for measurement was pregnancy timing and preferences. Measures such as the Reproductive Decision‐making measure (Hinson et al. 2019) and the Women's and Girl's Empowerment–SRH Index (Moreau et al. 2020) included items that measured the number of children desired and the spacing of these births. Many of these measures were focused on assessing agentive action about fertility. For instance, the Human Development Survey (Samanta 2020) includes questions about who has the final say on the number of children, and the Reproductive Autonomy Scale (Loll et al. 2019) asks who makes decisions on when to have children. All but one of these measures—Gender Behavior in Reproductive Health Scale (Yang et al. 2009)—were developed to measure fertility among either men or women. The Gender Behavior in Reproductive Health Scale was designed with two versions, each tailored to measure fertility among men and women separately.

Condom use was a less common focal area and measures focused on assessing this focal area within the realm of sexually transmitted infections and HIV prevention (Wagner et al. 2014) or assessed condom use together with contraceptive use (Agha et al. 2021, 2020). Apart from the aforementioned domains, generalized measures were not specifically tailored to a family planning or reproductive health domain and were classified in the “Other” category. These measures assessed the efficacy and agentive action related to the household, individual or couple finances, health‐related choices, and childcare responsibilities.

## DISCUSSION

Our review aimed to assess how agency has been measured across contexts in peer‐reviewed and gray family planning‐focused literature. We identified 58 unique measures of agency or related constructs, of which 44 of these measures included items tailored to family planning or reproductive health. These unique measures were implemented largely in women/girls, within Sub‐Saharan African and South Asian contexts, and included in studies published in 2016 or later. Measures focused nearly exclusively on the “can” and “act” components of agency, and most commonly included social norms when assessing a related construct of agency. Our findings demonstrate that measurement of agency constructs within family planning has varied across studies and focal areas, with a predominant focus on contraceptive use. While many measures solely assessed contraceptive use, some also assessed discussion of family planning, access to services, sexual relations, or fertility timing and preferences. This underscores the importance of expanding our measurement of agency and its related constructs beyond just family planning use. By broadening our scope to include other aspects of reproductive health, such as fertility and sexual relations, we can gain a more comprehensive understanding of an individual's reproductive agency. However, despite this recognition, there are significant gaps in measuring agentive action, efficacy, backlash to agentive action, and the related agency constructs within family planning, fertility, and sexual relations.

First, there were clear gaps in coverage in terms of measure attributes. First, several measures failed to capture the multifaceted nature of agency. While more than three‐fourths of identified studies used summative scores to construct family planning‐related agency measures, eight used dichotomous or categorical measures of agency. These categorical approaches, while methodologically simpler, often oversimplify the intricate interplay of psychological factors, agentive actions, and social or other structural factors that together capture the concept of agency (Bhan et al. 2020). The use of summative measures offers more opportunity for nuance, with consideration of different dimensions of agency such as decision‐making autonomy, negotiation power, and access to resources. However, most of these summative measures were not comprehensively representative of family planning‐related agency and fell short in determining how individuals came to those family planning‐related decisions, or whether they can, for example, persevere in acting upon them in the face of barriers. Fundamentally, for several measures, the reliance on dichotomous measures introduces significant limitations. These measures inherently reduce agency to a yes‐or‐no framework, whereby we may accept a measure of family planning decision‐making “independently” as sufficient to assert empowerment. However, this approach overlooks the spectrum of partial or shared decision‐making processes leading up to that act, the degree of influence exerted by external actors, or the individual's perceived versus actual control in decision‐making scenarios.

Addressing these gaps, and adopting a broader view of agency and its constructs, requires developing and employing multidimensional tools that can more effectively capture its complexity within family planning. Mixed‐method approaches, integrating quantitative scales with qualitative insights, may hold potential. Measures used to assess aspects of agency within family planning contexts should be tailored to that context and tested for salience, reliability, and validity (Holt et al. 2024). Additionally, as possible, measures should consider the temporal and situational fluidity of agency across family planning behaviors. For instance, in recognizing that agency is not stagnant, measures development studies should strive to understand agency in family planning along the different time points of the life course (e.g., prior to marriage, prior to first childbirth, after achievement of desired family size, as a new wife, as a mother‐in‐law) and develop and test measures among populations at these time points to assess the relevance and stability of a given measure over time. This would offer a substantial advance by recognizing that agency in family planning is not a static construct but one that evolves in response to changing personal and environmental factors.

Second, in the studies that captured the multifaceted nature of agency through multi‐item measures and summative construction, a significant portion failed to conduct psychometric evaluations, which are critical to validating the measures. Of the identified measures of agency, only 16 included assessments of psychometric reliability and validity. This presents a concern because psychometric testing plays a pivotal role in ensuring research rigor and quality, as it assesses the credibility and validity of measures (Cooper et al. 2017; Wilkinson and Task Force on Statistical Inference 1999). These tests examine the internal consistency of items including how they function cohesively to represent the underlying construct as well as the extent to which the measure accurately reflects the theoretical dimensions it is intended to capture. Importantly, psychometric properties are context‐sensitive and can vary significantly across populations, cultural settings, and time periods. For example, a family planning measure of agency that performs well in one geographic region or demographic group may not hold the same validity in another, due to differences in social norms, linguistic interpretations, or sociopolitical influences. Additionally, psychometric properties are not stagnant and can vary across contexts. Therefore, failing to report performing psychometric testing might bring the contextual measure performance of the remaining measures into question, particularly when using existing measures within different populations (Cooper et al. 2017).

Consistent with prior research (Bhan et al. 2020), this review identified very few measures of agency used in measuring family planning outcomes that focused on the “resist” construct of agency. In many contexts, family planning choices are not made in isolation but are influenced by complex power dynamics manifested in various ways. For instance, partners in intimate relationships may exert control over contraceptive choices (Sanchez et al. 2020) and the type of method used such as male‐controlled methods, female‐controlled methods, covert methods or overt methods. An individual may face coercion or pressure from partners, parents, family members, and others in the community to conform to certain contraceptive choices or practices (Sanchez et al. 2020; Chola, Hlongwana, and Ginindza 2023; Mutumba, Wekesa, and Stephenson 2018). Therefore, understanding the manifestations and consequences of an individual's ability to persist in their choice despite the external pressures is vital in addressing barriers to contraceptive autonomy and choice.

Furthermore, our findings highlight the gaps in measures of related elements that influence agency. While there were several measures on social norms, minimal attention was given to conviction, aspiration, and critical consciousness. Even within identified social norm measures, there is a need for greater focus on the specific components of social norms around family planning. Although some measures focused on measuring descriptive norms and injunctive norms, none assessed positive or negative sanctions for when the norm is violated. Social norms are often supported by sanctions and deviations from the behavioral standard may be punished (Bicchieri 2016), particularly when an individual's contraceptive needs or fertility desires go against these norms. For example, in societies with a high value on having many children, social norms may discourage contraceptive use and individuals who challenge these norms have the potential to face social sanctions.

Third, findings also highlight the dearth of agency measures designed for and used among men, restricting our understanding of the role of men's agency in family planning research and programming. In many settings, men often hold considerable influence over contraceptive choices including contraceptive use, and access to family planning services (Sanchez et al. 2020). Therefore, focusing primarily on women's agency, we could overlook the complex power dynamics that men may hold within contraceptive choices in relationships. Understanding men's agency is vital, particularly, amidst the growing emphasis on strategies advocating for male engagement in family planning as clients, supportive partners, and agents of change (DeGraw and Rottach 2021).

In addition to the lack of attention to men's agency in family planning, measures of agency were most commonly used in populations 18–29 years of age. Only 50 percent of measures assessing “can” or “resist” were used in populations under 18 years of age. These younger women face distinct social and cultural barriers when making family planning decisions or achieving their family planning goals. For example, younger women, particularly unmarried ones, may experience heightened pressure stemming from social norms, and familial expectations, which together with legal restrictions, may shape their sense of agency related to a myriad of family planning behaviors including contraceptive use and access (Bearinger et al. 2007). Expanding the scope of measuring agency within family planning to include younger, unmarried women is therefore essential for ensuring that programs are inclusive, responsive to diverse needs, and able to address the complex barriers to family planning‐related agency faced by all women, regardless of age or marital status.

Fourth, geographically, there's an overrepresentation of measures in sub‐Saharan Africa and South Asia while very little on measures in Central Asia, East Asia and Pacific, and Latin America and the Caribbean, restricting our understanding of agency measurement within these regions. Moreover, agency can be context‐specific and as such can vary across regions, emphasizing the importance of considering geographic or regional nuances. For instance, in some settings in South Asia, social norms and household structures may limit women's decision‐making power regarding contraceptive use, requiring negotiation with family members such as husbands or mothers‐in‐law (Jejeebhoy and Sathar 2024). In contrast, in parts of Latin America, where women's autonomy in reproductive health decision‐making has been shaped by different legal and social movements, participation in family planning decisions may be more common (Jejeebhoy and Sathar 2024). This context‐specific nature of agency highlights the need for caution when adapting measures of agency and emphasizes that adaptation of measures from one region to another may not always be appropriate (Edmeades et al. 2018).

Taken together, the results of this analysis suggest first and foremost that there are many promising measures available for use. These measures offer valuable insights into various aspects of agency related to family planning and reproductive health. Many of the tools, such as the Family Planning Self‐Efficacy Scale (Okigbo et al., 2018), the Reproductive Empowerment Scale (Mandal and Albert 2020), and the Reproductive Autonomy Scale (Loll et al., 2019), offer nuanced means of assessing aspects of self‐efficacy, decision‐making, and autonomy in family planning contexts. Measures like the Women's and Girl's Empowerment–SRH Index (Moreau et al., 2020) and the Contraceptive Self‐Efficacy Scale (McCarthy et al., 2019) contribute to understanding empowerment and self‐confidence in using contraception. Tools such as the Gender Norms scale (Sedlandar et al., 2023)  and the FP and Gender Transformative Decision‐Making scale (Wegs et al., 2016) are particularly useful for assessing the influence of social and gender norms on reproductive decisions. While these measures were often designed to focus on specific components of agency, they collectively can offer a more comprehensive view in aggregate. Nonetheless, a single measure that is programmatically relevant and feasible, and that fully captures the multiple constructs of family planning agency and related constructs that influence agency remains a gap in the field, and would provide even greater utility for family planning research and program development.

### Limitations

While this scoping review has many strengths, including a strong conceptual foundation and rigorous methodology, there are important limitations to note. We restricted our search to four electronic sources and the EMERGE database and in doing so, our review may have omitted relevant articles. The search was restricted to English publications; thus, we may have omitted agency measures developed or adapted in any other language. It is also plausible that the exclusion of non‐English literature could have limited our identification of the agency or related constructs that may have been studied in other languages. Additionally, the prevalence of sub‐Saharan Africa in the included studies may reflect differences in research priorities and measurement approaches across regions. South Asia has been central to the conceptualization of agency and empowerment as well as the measurement of these constructs. However, many studies in South Asia may rely more on broader gender and empowerment indices as well as other preexisting measures rather than the standalone, unique family‐planning‐specific agency measures that were the focus of this review. This distinction in measurement approaches may have contributed to the lower representation of South Asian studies in our analysis.

Further, we limited our search to studies that included measure items and response options, which may have omitted agency measures that were not publicly available. There may be measures of agency being used in family‐planning‐focused research that have not been disseminated in peer‐reviewed or gray literature. Finally, while we analyzed related constructs identified in the literature found, they were not included in our search terms thus, we may have omitted literature with measures focused solely on the related constructs. All these limitations reduce our analysis pool. There may thus be additional measures that were not identified in this analysis.

## CONCLUSIONS

While there is increased interest in measuring family planning agency in low‐ and middle‐income settings, this review highlights several important gaps in measurement in critical constructs of agency, such as the lack of measures on backlash to agentive action (“resist”) and related constructs. These measures reflect an overarching need within the field of family planning to broaden our measurement of family planning‐related agency to capture the multifaceted nature of agency and related constructs across populations and geographical regions. Moreover, family planning‐related agency measures should consider related constructs that situate agency within broader, multidimensional empowerment processes, including measures focused on awareness of choice, informed and free choice, and the achievement of self‐ and collective‐determined goals. Addressing the measurement gaps identified by this review will be instrumental in designing and evaluating empowerment‐focused programs and tracking progress, as well as informing family planning research, program, and policy development.

## CONFLICT OF INTEREST STATEMENT

The authors report no conflict of interest.

## ETHICS APPROVAL STATEMENT

This scoping review focused on published research as such, ethical approval was not required.

## PERMISSION TO REPRODUCE MATERIAL FROM OTHER SOURCES

We did not reproduce material from other sources.

## Supporting information



Appendix

## Data Availability

Studies included in the scoping review are available in the Supporting Information section.
