# Supplementary material for: Agency in Family Planning: A Scoping Review of the Measurement of Agency in Low‐ and Middle‐Income Countries
Source: Stud Fam Plann. 2025 Jul 9;56(3):621–41. doi: 10.1111/sifp.70025 (PMC12501686; doi:10.1111/sifp.70025)
Supplement: Supplementary file 1 — Appendix [file SIFP-56-621-s001.docx]

**Agency in Family Planning: A scoping review of the measurement of agency in low- and middle-income countries**

Authors: Francine Wood, Courtney McLarnon, Sarah Smith, Nitya Yerabandi, Lotus McDougal

# **Appendix**

**SEARCH STRATEGY**

**TABLE T1 Search Strategy for PubMed Database**

| **Concept** | **List of terms** |
| --- | --- |
| 1 - agency | (Empowerment[mesh] OR "personal autonomy"[mesh] or "patient participation"[mesh] or "self efficacy"[mesh]) OR (Whistleblowing[mesh]) OR ("social change"[mesh]) OR ("decision making"[mesh] or "negotiating"[mesh]) OR (empowerment[tw] or agency[tw] or "self efficacy"[tw]) OR ("decision-making"[tw] or "power to"[tw] or negotiati*[tw] or "patient participation"[tw]) OR ("social change"[tw] or "movement-building"[tw] or "social movement"[tw] or "collective power"[tw] or "collective agency"[tw] or "grassroots movement"[tw] or activism[tw]) |
| 2 - measurement | (“Dimensional measurement accuracy”[mesh]) OR (Measurement[tw] or conceptualization[tw] or valid*[tw]) |
| 3 – health domains: sexual and reproductive health, maternal and child health, quality of health care services, relationships, HIV/AIDS, COVID-19, nutrition, mental health, behavior change, multimedia, gender transformation, intimate partner violence | SRH[tw] OR (Health[tw] AND (sexual[tw] or “sexual and reproductive”[tw] or reproductive[tw] or adolescent[tw] or maternal[tw] or neonatal[tw] or child[tw])) OR (contraception[mesh] or “family planning services”[mesh] or contraception[tw] or “family planning”[tw] or “birth control”[tw] or “long-acting contraceptive method”[tw]) OR (HIV[tw] or “human immunodeficiency virus”[tw] or AIDS[tw] or “acquired immunodeficiency syndrome”[tw] or COVID-19[tw]) OR (“well-being”[tw] or “psychosocial health”[tw] or “mental health”[tw] or “psychological distress”[tw] or “mental distress”[tw] or anxiety[tw] or depression[tw] or PTSD[tw] or “health and well-being”[tw]) OR (“respectful care”[tw] or “quality of care”[tw] or “medical mistreatment”[tw]) OR (“marital choice”[tw] or “marriage decision-making”[tw] or “arranged marriage”[tw] or “household decision-making”[tw] or “household”[tw] or “intimate partner”[tw]) OR (Nutrition[tw] or “food consumption”[tw] or “feeding behavior”[tw] or “food literacy”[tw]) OR (Friendship[tw] or “social relationship”[tw] or “social integration”[tw] or “social interaction”[tw]) OR (“behavioral change”[tw] or “social change”[tw]) OR (multimedia[mesh] or “communications media”[mesh] or multimedia[tw] or “communications media”[tw]) OR gender transform*[tw] OR (“Intimate partner violence”[mesh] or “gender-based violence”[mesh] or GBV[tw] or IPV[tw] or “intimate partner violence”[tw] or “domestic violence”[tw] or “spousal abuse”[tw] or “marital abuse”[tw] or “marital violence”[tw] or “dating violence”[tw]) |
| 4 – Low- and middle-income countries | LMIC or development context* or “low and middle income countries” OR (upper middle income countr* or middle income countr* or (Albania or American Samoa or Argentina or Armenia or Azerbaijan or Belarus or Belize or “Bosnia and Herzegovina” or Bosnia or Bostwana or Brazil or Bulgaria or China or Colombia or “Costa Rica” or Cuba or Dominica or “Dominican Republic” or Ecuador or Fiji or Gabon or Georgia or Grenada or “Equatorial Guinea” or Guatemala or Guyana or Iraq or Jamaica or Jordan or Kazakhstan or Kosovo or Libya or St. Lucia or Malaysia or Maldives or “Marshall Islands” or Mauritius or Mexico or Moldova or Montenegro or Namibia or “North Macedonia” or Palau or Paraguay or Peru or Russia or “Russian Federation” or Serbia or “South Africa” or Suriname or Thailand or Tonga or Turkmenistan or Turkey or Türkiye or  Tuvalu or “St. Vincent and the Grenadines” or “St. Vincent”)) OR (Lower-middle income countr* OR (Algeria or Angola or Bangladesh or Benin or Bhutan or Bolivia or “Cabo Verde” or Cambodia or Cameroon or “Côte d’Ivoire” or Comoros or “Republic of the Congo” or Djibouti or Egypt or “El Salvador” or Eswatini or Ghana or Haiti or Honduras or India or Indonesia or Iran or Kenya or Kiribati or “Kyrgyz Republic” or Laos or Lebanon or Lesotho or Mauritania or Micronesia or Mongolia or Morocco or Myanmar or Nepal or Nicaragua or Nigeria or Pakistan or “Papua New Guinea” or Philippines or Samoa or Senegal or “Solomon Islands” or “Sri Lanka” or “São Tomé and Príncipe” or “West Bank” or Gaza or Tajikistan or Tanzania or Timor-Leste or Tunisia or Ukraine or Uzbekistan or Vietnam or Vanuatu or Zimbabwe)) OR (Low-income countr* OR (Afghanistan or Burkina Faso or Burundi or “Central African Republic” or Chad or “Democratic Republic of the Congo” or DRC or Ethiopia or Eritrea or Gambia or Guinea or Guinea-Bissau or Liberia or Madagascar or Malawi or Mali or Mozambique or Niger or “North Korea” or Rwanda or Sudan or “Sierra Leone” or Somalia or “South Sudan” or “Syrian Arab Republic” or Togo or Uganda or Yemen or Zambia)) |
| All concepts combined | (("Dimensional measurement accuracy"[MeSH Terms] OR ("Measurement"[Text Word] OR "conceptualization"[Text Word] OR "valid*"[Text Word])) AND 2000/01/01:2022/11/30[Date - Publication] AND (("empowerment"[MeSH Terms] OR "personal autonomy"[MeSH Terms] OR "patient participation"[MeSH Terms] OR "self efficacy"[MeSH Terms] OR "whistleblowing"[MeSH Terms] OR "social change"[MeSH Terms] OR ("decision-making"[MeSH Terms] OR "negotiating"[MeSH Terms]) OR ("empowerment"[Text Word] OR "agency"[Text Word] OR "self efficacy"[Text Word]) OR ("decision-making"[Text Word] OR "power to"[Text Word] OR "negotiati*"[Text Word] OR "patient participation"[Text Word]) OR ("social change"[Text Word] OR "movement-building"[Text Word] OR "social movement"[Text Word] OR "collective power"[Text Word] OR "collective agency"[Text Word] OR "grassroots movement"[Text Word] OR "activism"[Text Word])) AND 2000/01/01:2022/11/30[Date - Publication]) AND (("LMIC"[All Fields] OR (("develop"[All Fields] OR "develope"[All Fields] OR "developed"[All Fields] OR "developer"[All Fields] OR "developer s"[All Fields] OR "developers"[All Fields] OR "developing"[All Fields] OR "developments"[All Fields] OR "develops"[All Fields] OR "growth and development"[MeSH Subheading] OR ("growth"[All Fields] AND "development"[All Fields]) OR "growth and development"[All Fields] OR "development"[All Fields]) AND "context*"[All Fields]) OR "low and middle income countries"[All Fields] OR ((("upper"[All Fields] OR "uppers"[All Fields]) AND ("middle"[All Fields] OR "middles"[All Fields]) AND ("income"[MeSH Terms] OR "income"[All Fields] OR "incomes"[All Fields] OR "income s"[All Fields]) AND "countr*"[All Fields]) OR (("middle"[All Fields] OR "middles"[All Fields]) AND ("income"[MeSH Terms] OR "income"[All Fields] OR "incomes"[All Fields] OR "income s"[All Fields]) AND "countr*"[All Fields]) OR ("albania"[MeSH Terms] OR "albania"[All Fields] OR ("american samoa"[MeSH Terms] OR ("american"[All Fields] AND "samoa"[All Fields]) OR "american samoa"[All Fields]) OR ("argentina"[MeSH Terms] OR "argentina"[All Fields] OR "argentina s"[All Fields] OR "argentinae"[All Fields]) OR ("armenia"[MeSH Terms] OR "armenia"[All Fields]) OR ("azerbaijan"[MeSH Terms] OR "azerbaijan"[All Fields]) OR ("republic of belarus"[MeSH Terms] OR ("republic"[All Fields] AND "belarus"[All Fields]) OR "republic of belarus"[All Fields] OR "belarus"[All Fields]) OR ("belize"[MeSH Terms] OR "belize"[All Fields]) OR "Bosnia and Herzegovina"[All Fields] OR ("Bosnia and Herzegovina"[MeSH Terms] OR ("bosnia"[All Fields] AND "herzegovina"[All Fields]) OR "Bosnia and Herzegovina"[All Fields] OR "bosnia"[All Fields]) OR "Bostwana"[All Fields] OR ("brazil"[MeSH Terms] OR "brazil"[All Fields] OR "brazil s"[All Fields] OR "brazils"[All Fields]) OR ("bulgaria"[MeSH Terms] OR "bulgaria"[All Fields]) OR ("china"[MeSH Terms] OR "china"[All Fields] OR "china s"[All Fields] OR "chinas"[All Fields]) OR ("colombia"[MeSH Terms] OR "colombia"[All Fields] OR "colombia s"[All Fields]) OR "Costa Rica"[All Fields] OR ("cuba"[MeSH Terms] OR "cuba"[All Fields]) OR ("dominica"[MeSH Terms] OR "dominica"[All Fields]) OR "Dominican Republic"[All Fields] OR ("ecuador"[MeSH Terms] OR "ecuador"[All Fields] OR "ecuador s"[All Fields]) OR ("fiji"[MeSH Terms] OR "fiji"[All Fields]) OR ("gabon"[MeSH Terms] OR "gabon"[All Fields]) OR ("georgia"[MeSH Terms] OR "georgia"[All Fields] OR "georgia republic"[MeSH Terms] OR ("georgia"[All Fields] AND "republic"[All Fields]) OR "georgia republic"[All Fields] OR "georgia s"[All Fields]) OR ("grenada"[MeSH Terms] OR "grenada"[All Fields]) OR "Equatorial Guinea"[All Fields] OR ("guatemala"[MeSH Terms] OR "guatemala"[All Fields] OR "guatemala s"[All Fields]) OR ("guyana"[MeSH Terms] OR "guyana"[All Fields]) OR ("iraq"[MeSH Terms] OR "iraq"[All Fields]) OR ("jamaica"[MeSH Terms] OR "jamaica"[All Fields] OR "jamaica s"[All Fields]) OR ("jordan"[MeSH Terms] OR "jordan"[All Fields]) OR ("kazakhstan"[MeSH Terms] OR "kazakhstan"[All Fields] OR "kazakhstan s"[All Fields]) OR ("kosovo"[MeSH Terms] OR "kosovo"[All Fields] OR "kosovo s"[All Fields]) OR ("libya"[MeSH Terms] OR "libya"[All Fields]) OR ("saint lucia"[MeSH Terms] OR ("saint"[All Fields] AND "lucia"[All Fields]) OR "saint lucia"[All Fields] OR ("st"[All Fields] AND "lucia"[All Fields]) OR "st lucia"[All Fields]) OR ("malaysia"[MeSH Terms] OR "malaysia"[All Fields] OR "malaysia s"[All Fields]) OR ("indian ocean islands"[MeSH Terms] OR ("indian"[All Fields] AND "ocean"[All Fields] AND "islands"[All Fields]) OR "indian ocean islands"[All Fields] OR "maldives"[All Fields] OR "maldive"[All Fields]) OR "Marshall Islands"[All Fields] OR ("mauritius"[MeSH Terms] OR "mauritius"[All Fields]) OR ("mexico"[MeSH Terms] OR "mexico"[All Fields] OR "mexico s"[All Fields] OR "mexicos"[All Fields]) OR ("moldova"[MeSH Terms] OR "moldova"[All Fields]) OR ("montenegro"[MeSH Terms] OR "montenegro"[All Fields]) OR ("namibia"[MeSH Terms] OR "namibia"[All Fields] OR "namibia s"[All Fields]) OR "North Macedonia"[All Fields] OR ("palau"[MeSH Terms] OR "palau"[All Fields] OR "palau s"[All Fields]) OR ("paraguai"[All Fields] OR "paraguay"[MeSH Terms] OR "paraguay"[All Fields]) OR ("peru"[MeSH Terms] OR "peru"[All Fields]) OR ("russia"[MeSH Terms] OR "russia"[All Fields] OR "russia s"[All Fields] OR "russias"[All Fields]) OR "Russian Federation"[All Fields] OR ("serbia"[MeSH Terms] OR "serbia"[All Fields]) OR "South Africa"[All Fields] OR ("suriname"[MeSH Terms] OR "suriname"[All Fields] OR "surinam"[All Fields]) OR ("thailand"[MeSH Terms] OR "thailand"[All Fields] OR "thailand s"[All Fields]) OR ("tonga"[MeSH Terms] OR "tonga"[All Fields] OR "tonga s"[All Fields]) OR ("turkmenistan"[MeSH Terms] OR "turkmenistan"[All Fields]) OR ("turkey"[MeSH Terms] OR "turkey"[All Fields] OR "turkey s"[All Fields] OR "turkeys"[MeSH Terms] OR "turkeys"[All Fields]) OR "Turkiye"[All Fields] OR ("micronesia"[MeSH Terms] OR "micronesia"[All Fields] OR "tuvalu"[All Fields]) OR "st vincent and the grenadines"[All Fields] OR "st vincent"[All Fields])) OR (("Lower-middle"[All Fields] AND ("income"[MeSH Terms] OR "income"[All Fields] OR "incomes"[All Fields] OR "income s"[All Fields]) AND "countr*"[All Fields]) OR ("algeria"[MeSH Terms] OR "algeria"[All Fields] OR ("angola"[MeSH Terms] OR "angola"[All Fields] OR "angola s"[All Fields]) OR ("bangladesh"[MeSH Terms] OR "bangladesh"[All Fields] OR "bangladesh s"[All Fields]) OR ("benin"[MeSH Terms] OR "benin"[All Fields] OR "benin s"[All Fields]) OR ("bhutan"[MeSH Terms] OR "bhutan"[All Fields] OR "bhutan s"[All Fields]) OR ("bolivia"[MeSH Terms] OR "bolivia"[All Fields]) OR "Cabo Verde"[All Fields] OR ("cambodia"[MeSH Terms] OR "cambodia"[All Fields] OR "cambodia s"[All Fields]) OR ("cameroon"[MeSH Terms] OR "cameroon"[All Fields] OR "cameroons"[All Fields] OR "cameroon s"[All Fields]) OR "Cote d'Ivoire"[All Fields] OR ("comoros"[MeSH Terms] OR "comoros"[All Fields] OR "comoro"[All Fields]) OR "Republic of the Congo"[All Fields] OR ("djibouti"[MeSH Terms] OR "djibouti"[All Fields]) OR ("egypt"[MeSH Terms] OR "egypt"[All Fields] OR "egypt s"[All Fields]) OR "El Salvador"[All Fields] OR ("eswatini"[MeSH Terms] OR "eswatini"[All Fields]) OR ("ghana"[MeSH Terms] OR "ghana"[All Fields] OR "ghana s"[All Fields]) OR ("haiti"[MeSH Terms] OR "haiti"[All Fields] OR "haiti s"[All Fields]) OR ("honduras"[MeSH Terms] OR "honduras"[All Fields]) OR ("india"[MeSH Terms] OR "india"[All Fields] OR "india s"[All Fields] OR "indias"[All Fields]) OR ("indonesia"[MeSH Terms] OR "indonesia"[All Fields] OR "indonesia s"[All Fields] OR "indonesias"[All Fields]) OR ("iran"[MeSH Terms] OR "iran"[All Fields]) OR ("kenya"[MeSH Terms] OR "kenya"[All Fields] OR "kenya s"[All Fields]) OR ("micronesia"[MeSH Terms] OR "micronesia"[All Fields] OR "kiribati"[All Fields]) OR "Kyrgyz Republic"[All Fields] OR ("laos"[MeSH Terms] OR "laos"[All Fields]) OR ("lebanon"[MeSH Terms] OR "lebanon"[All Fields] OR "lebanon s"[All Fields]) OR ("lesotho"[MeSH Terms] OR "lesotho"[All Fields]) OR ("mauritania"[MeSH Terms] OR "mauritania"[All Fields]) OR ("micronesia"[MeSH Terms] OR "micronesia"[All Fields]) OR ("mongolia"[MeSH Terms] OR "mongolia"[All Fields] OR "mongolia s"[All Fields]) OR ("morocco"[MeSH Terms] OR "morocco"[All Fields]) OR ("myanmar"[MeSH Terms] OR "myanmar"[All Fields] OR "myanmar s"[All Fields] OR "myanmars"[All Fields]) OR ("nepal"[MeSH Terms] OR "nepal"[All Fields] OR "nepal s"[All Fields]) OR ("nicaragua"[MeSH Terms] OR "nicaragua"[All Fields] OR "nicaragua s"[All Fields]) OR ("nigeria"[MeSH Terms] OR "nigeria"[All Fields] OR "nigeria s"[All Fields]) OR ("pakistan"[MeSH Terms] OR "pakistan"[All Fields] OR "pakistan s"[All Fields]) OR "Papua New Guinea"[All Fields] OR ("philippine"[All Fields] OR "philippines"[MeSH Terms] OR "philippines"[All Fields]) OR ("samoa"[MeSH Terms] OR "samoa"[All Fields] OR "samoas"[All Fields]) OR ("senegal"[MeSH Terms] OR "senegal"[All Fields] OR "senegal s"[All Fields]) OR "Solomon Islands"[All Fields] OR "Sri Lanka"[All Fields] OR "Sao Tome and Principe"[All Fields] OR "West Bank"[All Fields] OR "Gaza"[All Fields] OR ("tajikistan"[MeSH Terms] OR "tajikistan"[All Fields]) OR ("tanzania"[MeSH Terms] OR "tanzania"[All Fields] OR "tanzania s"[All Fields]) OR ("timor leste"[MeSH Terms] OR "timor leste"[All Fields] OR ("timor"[All Fields] AND "leste"[All Fields]) OR "timor leste"[All Fields]) OR ("tunisia"[MeSH Terms] OR "tunisia"[All Fields]) OR ("ukraine"[MeSH Terms] OR "ukraine"[All Fields] OR "ukraine s"[All Fields]) OR ("uzbekistan"[MeSH Terms] OR "uzbekistan"[All Fields]) OR ("vietnam"[MeSH Terms] OR "vietnam"[All Fields] OR "vietnam s"[All Fields]) OR ("vanuatu"[MeSH Terms] OR "vanuatu"[All Fields]) OR ("zimbabwe"[MeSH Terms] OR "zimbabwe"[All Fields] OR "zimbabwe s"[All Fields]))) OR ((("poverty"[MeSH Terms] OR "poverty"[All Fields] OR ("low"[All Fields] AND "income"[All Fields]) OR "low income"[All Fields]) AND "countr*"[All Fields]) OR ("afghanistan"[MeSH Terms] OR "afghanistan"[All Fields] OR "afghanistan s"[All Fields] OR ("burkina faso"[MeSH Terms] OR ("burkina"[All Fields] AND "faso"[All Fields]) OR "burkina faso"[All Fields]) OR ("burundi"[MeSH Terms] OR "burundi"[All Fields]) OR "Central African Republic"[All Fields] OR ("chad"[MeSH Terms] OR "chad"[All Fields]) OR "Democratic Republic of the Congo"[All Fields] OR "DRC"[All Fields] OR ("ethiopia"[MeSH Terms] OR "ethiopia"[All Fields] OR "ethiopia s"[All Fields]) OR ("eritrea"[MeSH Terms] OR "eritrea"[All Fields]) OR ("gambia"[MeSH Terms] OR "gambia"[All Fields] OR "gambia s"[All Fields]) OR ("guinea"[MeSH Terms] OR "guinea"[All Fields] OR "guinea s"[All Fields] OR "guineas"[All Fields]) OR ("guinea bissau"[MeSH Terms] OR "guinea bissau"[All Fields] OR ("guinea"[All Fields] AND "bissau"[All Fields]) OR "guinea bissau"[All Fields]) OR ("liberia"[MeSH Terms] OR "liberia"[All Fields] OR "liberia s"[All Fields]) OR ("madagascar"[MeSH Terms] OR "madagascar"[All Fields] OR "madagascar s"[All Fields]) OR ("malawi"[MeSH Terms] OR "malawi"[All Fields] OR "malawi s"[All Fields]) OR ("mali"[MeSH Terms] OR "mali"[All Fields]) OR ("mozambique"[MeSH Terms] OR "mozambique"[All Fields] OR "mozambique s"[All Fields]) OR ("niger"[MeSH Terms] OR "niger"[All Fields]) OR "North Korea"[All Fields] OR ("rwanda"[MeSH Terms] OR "rwanda"[All Fields] OR "rwanda s"[All Fields]) OR ("sudan"[MeSH Terms] OR "sudan"[All Fields] OR "sudans"[All Fields] OR "sudan s"[All Fields]) OR "Sierra Leone"[All Fields] OR ("somalia"[MeSH Terms] OR "somalia"[All Fields]) OR "South Sudan"[All Fields] OR "Syrian Arab Republic"[All Fields] OR ("togo"[MeSH Terms] OR "togo"[All Fields]) OR ("uganda"[MeSH Terms] OR "uganda"[All Fields] OR "uganda s"[All Fields]) OR ("yemen"[MeSH Terms] OR "yemen"[All Fields]) OR ("zambia"[MeSH Terms] OR "zambia"[All Fields] OR "zambia s"[All Fields])))) AND 2000/01/01:2022/11/30[Date - Publication]) AND (("SRH"[Text Word] OR ("Health"[Text Word] AND ("sexual"[Text Word] OR "sexual and reproductive"[Text Word] OR "reproductive”[Text Word] OR "adolescent"[Text Word] OR "maternal"[Text Word] OR "neonatal"[Text Word] OR "child"[Text Word])) OR ("contraception"[MeSH Terms] OR "family planning services"[MeSH Terms] OR "contraception"[Text Word] OR "family planning"[Text Word] OR "birth control"[Text Word] OR "long-acting contraceptive method"[Text Word]) OR ("HIV"[Text Word] OR "human immunodeficiency virus"[Text Word] OR "AIDS"[Text Word] OR "acquired immunodeficiency syndrome"[Text Word] OR "COVID-19"[Text Word]) OR ("well-being"[Text Word] OR "psychosocial health"[Text Word] OR "mental health"[Text Word] OR "psychological distress"[Text Word] OR "mental distress"[Text Word] OR "anxiety"[Text Word] OR "depression"[Text Word] OR "PTSD"[Text Word] OR "health and well-being"[Text Word]) OR ("respectful care"[Text Word] OR "quality of care"[Text Word] OR "medical mistreatment"[Text Word]) OR ("marital choice"[Text Word] OR "marriage decision-making"[Text Word] OR "arranged marriage"[Text Word] OR "household decision-making"[Text Word] OR "household"[Text Word] OR "intimate partner"[Text Word]) OR ("Nutrition"[Text Word] OR "food consumption"[Text Word] OR "feeding behavior"[Text Word] OR "food literacy"[Text Word]) OR ("Friendship"[Text Word] OR "social relationship"[Text Word] OR "social integration"[Text Word] OR "social interaction"[Text Word]) OR ("behavioral change"[Text Word] OR "social change"[Text Word]) OR ("multimedia"[MeSH Terms] OR "communications media"[MeSH Terms] OR "multimedia"[Text Word] OR "communications media"[Text Word]) OR "gender transform*"[Text Word] OR ("Intimate partner violence"[MeSH Terms] OR "gender-based violence"[MeSH Terms] OR "GBV"[Text Word] OR "IPV"[Text Word] OR "Intimate partner violence"[Text Word] OR "domestic violence"[Text Word] OR "spousal abuse"[Text Word] OR "marital abuse"[Text Word] OR "marital violence"[Text Word] OR "dating violence"[Text Word])) AND 2000/01/01:2022/11/30[Date - Publication])) AND (2000/1/1:2022/11/30[pdat]) |

Note: All searches were restricted to January 1 2000 – November 30, 2022.

**TABLE T2 Search Strategy for CINAHL Full-Text and PsycINFO Databases, using EBSCOHost**

| **Concept** | **List of terms** |
| --- | --- |
| 1 - agency | AB Empowerment OR AB personal autonomy OR AB patient participation OR AB ( self-efficacy or self efficacy or confidence ) OR AB individual agency OR AB whistleblowing OR AB ( social change or social justice ) OR AB negotiation OR AB collective power OR AB grassroots movements OR AB collective agency |
| 2 - measurement | AB dimensional measure OR AB conceptual understanding OR AB ( measure or scale ) OR AB ( validation or validity or validation studies ) |
| 3 – health domains: sexual and reproductive health, maternal and child health, quality of health care services, relationships, HIV/AIDS, COVID-19, nutrition, mental health, behavior change, multimedia, gender transformation, intimate partner violence | AB ( sexual and reproductive health or SRH ) OR AB ( contraception or birth control or family planning or contraceptive ) OR AB ( hiv or aids or acquired human immunodeficiency syndrome or human immunodeficiency virus ) OR AB covid-19 OR AB ( wellbeing or well-being or mental health ) OR AB ( decision making) OR AB ( behavior change ) OR AB ( gender transformative approach) OR AB ( multimedia ) OR AB ( intimate partner violence or domestic violence or partner abuse or intimate partner aggression or IPV or GBV or gender-based violence or marital abuse or spousal abuse or marital violence ) OR AB ( nutrition or food intake or feeding behavior or food literacy ) OR AB ( social support or social networks or social relationships or friendship ) OR AB ( quality of care or respectful care ) OR AB ( maternal and child health or neonatal health or adolescent health ) |
| 4 – Low- and middle-income countries | AB LMIC or AB ( developing countries ) or AB ( development context ) or AB ( low and middle income countries ) OR (upper middle income countr* or middle income countr* or (Albania or American Samoa or Argentina or Armenia or Azerbaijan or Belarus or Belize or “Bosnia and Herzegovina” or Bosnia or Bostwana or Brazil or Bulgaria or China or Colombia or “Costa Rica” or Cuba or Dominica or “Dominican Republic” or Ecuador or Fiji or Gabon or Georgia or Grenada or “Equatorial Guinea” or Guatemala or Guyana or Iraq or Jamaica or Jordan or Kazakhstan or Kosovo or Libya or St. Lucia or Malaysia or Maldives or “Marshall Islands” or Mauritius or Mexico or Moldova or Montenegro or Namibia or “North Macedonia” or Palau or Paraguay or Peru or Russia or “Russian Federation” or Serbia or “South Africa” or Suriname or Thailand or Tonga or Turkmenistan or Turkey or Türkiye or  Tuvalu or “St. Vincent and the Grenadines” or “St. Vincent”)) OR (Lower-middle income countr* OR (Algeria or Angola or Bangladesh or Benin or Bhutan or Bolivia or “Cabo Verde” or Cambodia or Cameroon or “Côte d’Ivoire” or Comoros or “Republic of the Congo” or Djibouti or Egypt or “El Salvador” or Eswatini or Ghana or Haiti or Honduras or India or Indonesia or Iran or Kenya or Kiribati or “Kyrgyz Republic” or Laos or Lebanon or Lesotho or Mauritania or Micronesia or Mongolia or Morocco or Myanmar or Nepal or Nicaragua or Nigeria or Pakistan or “Papua New Guinea” or Philippines or Samoa or Senegal or “Solomon Islands” or “Sri Lanka” or “São Tomé and Príncipe” or “West Bank” or Gaza or Tajikistan or Tanzania or Timor-Leste or Tunisia or Ukraine or Uzbekistan or Vietnam or Vanuatu or Zimbabwe)) OR (Low-income countr* OR (Afghanistan or Burkina Faso or Burundi or “Central African Republic” or Chad or “Democratic Republic of the Congo” or DRC or Ethiopia or Eritrea or Gambia or Guinea or Guinea-Bissau or Liberia or Madagascar or Malawi or Mali or Mozambique or Niger or “North Korea” or Rwanda or Sudan or “Sierra Leone” or Somalia or “South Sudan” or “Syrian Arab Republic” or Togo or Uganda or Yemen or Zambia)) |
| All search terms combined | 1 AND 2 AND 3 AND 4 |

Note: All searches were restricted to January 1 2000 – November 30 2022 in English, with search terms included in the article abstract.

**TABLE T3 Search Strategy for ProQuest Database**

| **Concept** | **List of terms** |
| --- | --- |
| 1 – population | TIAB("women") OR TIAB("girls") OR TIAB(“boys”) OR TIAB("child") OR TIAB("adolescent") OR TIAB("men") |
| 2 – agency | TIAB("agency") OR TIAB("empowerment") |
| 3 – measurement | TIAB("measure*") OR TIAB("valid*") |
| 4 – health domains | TIAB(“health”) |
| All search terms combined | (TIAB("women") OR TIAB("girls") OR TIAB(“boys”) OR TIAB("child") OR TIAB("adolescent") OR TIAB("men")) AND (TIAB("agency") OR TIAB("empowerment")) AND (TIAB("measure*") OR TIAB("valid*")) AND TIAB("health") |

Note: TIAB refers to ‘title or abstract’, and all searches were restricted to title and abstract to identify articles which may be most relevant to the scoping review. Article searches were also restricted to January 1 2000 – November 30 2022.

**TABLE T4 Search Strategy for Evidence-based Measures of Empowerment for Research on Gender and Equality (EMERGE) Website**

| **Concept** | **List of terms** |
| --- | --- |
| 1 – individual agency & measurement | Individual agency AND measure AND (health or wellbeing or HIV or COVID-19 or nutrition or psycholog* or social or behavior or IPV or GBV) |
| 2 – collective agency & measurement | Collective agency AND measure |

**Identified measures**

**TABLE T5 List of measures identified in the articles included in the scoping review**

|  | **# of times used** | **Construction of Measure** | **Psychometric Properties (if summative)** | **Agency Constructs** | **Related Construct** | **Level of Social Ecology** | **Sample** | **Measure includes items on Family Planning** | **Journal Article (Author, Year)** |
| --- | --- | --- | --- | --- | --- | --- | --- | --- | --- |
| AIDS Prevention Questionnaire | 1 | Categorical | n/a | Can |  | Individual | Women/Girls | Yes (four items) | (Giménez-García et al. 2018) |
| Condom use self-efficacy - Wagner | 1 | Summative | Not reported | Can |  | Individual | Both genders; Marginalized population | Yes (one item – only item in measure) | (Wagner et al. 2014) |
| Condom Use Self-Efficacy Scale | 1 | Summative | Reliability and validity | Can |  | Individual | Both genders | Yes (all 14 items) | (Asante and Doku 2010) |
| Contraception use intention - Hamid | 1 | Categorical | n/a |  | Aspiration or goal-setting | Individual | Women/Girls | Yes (one item – only item in measure) | (Hamid et al. 2011) |
| Contraception use intention - Sedlandar | 1 | Categorical | n/a |  | Aspiration or goal-setting | Individual | Women/Girls | Yes (one item – only item in measure) | (Sedlander et al. 2023) |
| Contraceptive and FP Norms | 1 | Categorical | n/a |  | Social Norms | Community | Both genders | Yes (all three items) | (Silva et al. 2021) |
| Contraceptive Self-efficacy - Kahsay | 1 | Categorical | n/a | Can |  | Individual | Women/Girls | Yes (all four items) | (Kahsay et al. 2018) |
| Contraceptive self-efficacy - Newmann | 1 | Summative | Reliability and validity | Can |  | Individual | Men/Boys | Yes (all three items) | (Newmann et al. 2021) |
| Contraceptive self-efficacy scale - Levinson | 3 | Summative | Reliability and validity (n=2), reliability (n=1) | Can |  | Individual | Women/Girls | Yes (all 18 items) | (Whiting-Collins et al. 2020; Arias et al. 2017; Whiting-Collins 2021) |
| Contraceptive self-efficacy scale - McCarthy | 1 | Categorical | n/a | Can | Conviction | Individual, Community | Women/Girls; Marginalized population | Yes (all six items) | (McCarthy et al. 2019) |
| Contraceptive subjective norms - Kahsay | 1 | Categorical | n/a |  | Social Norms | Individual, Community | Women/Girls | Yes (all four items) | (Kahsay et al. 2018) |
| Decision making - Hossain | 1 | Summative | Not reported | Act |  | Individual | Women/Girls | Yes (one item) | (Hossain and Kabir 2001) |
| Decision Making - National Survey of Household Characteristics | 1 | Summative | Reliability only | Act | Autonomy | Individual, Interpersonal | Both genders | None | (Feldman et al. 2009) |
| Decision Making - Tajikistan Living Standards Survey | 1 | Categorical | n/a | Act |  | Individual | Women/Girls | None | (Kamiya 2010) |
| Decision-making - Human Development Survey | 1 | Summative | Reliability and validity | Act |  | Individual | Women/Girls | Yes (one item) | (Samanta 2020) |
| Demographic and Health Survey | 26 | Summative | Reliability only (n=4), Validity only (n=4), Reliability and validity (n=5), Not reported (n=4) | Can, Act | Aspiration or goal-setting, Social Norms, Autonomy, Other | Individual, Interpersonal, Other | Women/Girls (n=24), Men/Boys (n=1) Both genders (n=1) | Yes (FP/RH measure items included 13 out of the 26 times DHS was used) | See Table A6 |
| Fertility control - Pande | 1 | Summative | Reliability and validity |  | Other | Individual, Interpersonal | Women/Girls | Yes (all three items) | (Pande et al. 2011) |
| Financial decision making - Panade | 1 | Summative | Reliability and validity | Act |  | Individual, Interpersonal | Women/Girls | None | (Pande et al. 2011) |
| Financial decision making - Singh | 1 | Summative | Not reported | Act |  | Individual | Women/Girls | None | (Singh et al. 2021) |
| Fogg Behavior Model | 2 | Summative | Reliability only (n=2) | Can | Aspiration or goal-setting, Social Norms, Motivation, Other | Individual, Interpersonal | Women/Girls | Yes (all 41 items) | (Agha et al. 2020; Agha et al. 2021) |
| FP and gender transformative decision making scale - Wegs | 1 | Summative | Reliability only | Act | Social Norms | Individual, Interpersonal | Both genders | Yes (two items) | (Wegs et al. 2016) |
| FP self-efficacy - Okigbo | 1 | Summative | Reliability only | Can, Resist |  | Individual | Women/Girls | Yes (all seven items) | (Okigbo et al. 2018) |
| FP tool - Alemayehu | 1 | Categorical |  | Can, Act |  | Individual, Interpersonal | Women/Girls | Yes (all 17 items) | (Alemayehu et al. 2020) |
| FP use self-efficacy | 1 | Summative | Reliability only | Can |  | Individual | Both genders | Yes (all four items) | (Wegs et al. 2016) |
| FP use/discussion self-efficacy | 1 | Summative | Not reported | Can, Resist |  | Individual | Women/Girls | Yes (all 20 items) | (Richardson 2016) |
| Freedom of movement - Hamid | 1 | Summative | Not reported | Act |  | Individual | Women/Girls | None | (Hamid et al. 2011) |
| Gender Behavior in Reproductive Health Scale | 1 | Summative | Reliability and validity |  | Other | Individual | Both genders | Yes (14 items each in men’s and women’s scale) | (Yang et al. 2009) |
| Gender norms - Paek | 1 | Summative | Reliability only |  | Social Norms | Community | Both genders | None | (Paek et al. 2008) |
| Gender norms - Singh | 1 | Summative | Not reported | Act | Social Norms | Individual | Women/Girls | Yes (two items) | (Singh et al. 2021) |
| Gender norms - Waszak | 1 | Categorical | n/a | Can | Social Norms | Individual, Interpersonal, Community | Women/Girls | None | (Waszak et al. 2001) |
| Gender norms scale | 1 | Summative | Reliability and validity | Act | Social Norms | Individual, Interpersonal, Community | Women/Girls | Yes (six items) | (Sedlander et al. 2023) |
| General Self-Efficacy Scale - Wagner | 1 | Summative | Not reported | Can |  | Individual | Both genders; Marginalized population | None | (Wagner et al. 2014) |
| Intention to use oral contraceptive tool | 1 | Summative | Reliability and validity |  | Social Norms | Community | Women/Girls | Yes (all eight items) | (Kridli and Libbus 2002) |
| Marital choice and agency - Hamid | 1 | Summative | Not reported | Act |  | Individual | Women/Girls | None | (Hamid et al. 2011) |
| Masculine Norms and Family Planning Acceptance (MNFPA) scale | 1 | Summative | Reliability and validity |  | Social Norms | Interpersonal | Men/Boys | Yes (all 10 items) | (Newmann et al. 2021) |
| Measurement, Learning and Evaluation Project | 1 | Summative | Validity only | Act | Social Norms | Individual, Interpersonal | Women/Girls | Yes (one item) | (Corroon et al. 2014) |
| Multidimensional Scale of Perceived Social Support | 1 | Summative | Reliability and validity |  | Social Support |  | Women/Girls | None | (Kiani et al. 2020) |
| Partner IUD discussion self-efficacy - Ha | 1 | Categorical | n/a | Can |  | Individual, Interpersonal | Men/Boys | Yes (all eight items) | (Ha et al. 2003) |
| Person-centered abortion care scale | 1 | Summative | Reliability and validity |  | Autonomy | Individual, Interpersonal | Women/Girls | Yes (one item – only item in measure) | (Sudhinaraset et al. 2018) |
| Person-centered care for family planning scale | 1 | Summative | Reliability and validity |  | Autonomy, Social Support | Individual, Interpersonal | Women/Girls | Yes (all six items) | (Sudhinaraset et al. 2020) |
| Pregnancy-Related Empowerment Scale | 1 | Summative | Reliability and validity | Can, Act |  | Individual, Interpersonal | Women/Girls | None (maternal health focused) | (Somji et al. 2022) |
| Reproductive Autonomy Scale | 1 | Summative | Reliability only | Act | Autonomy | Individual | Women/Girls | Yes (all 5 items) | (Loll et al. 2019) |
| Reproductive decision-making - Hindson | 1 | Summative | Reliability and validity | Act |  | Individual | Both genders | Yes (all 10 items) | (Hinson et al. 2019) |
| Reproductive Empowerment Scale | 1 | Summative | Reliability and validity | Can, Act | Critical Consciousness, Social Norms, Social Support | Individual, Interpersonal | Both genders | Yes (all 20 items) | (Mandal and Albert 2020) |
| Rosenberg Self-Esteem Scale | 1 | Summative | Reliability and validity | Act |  |  | Women/Girls | None | (Kiani et al. 2020) |
| Safer Sex Self-Efficacy Scale | 1 | Categorical |  | Act |  | Individual, Interpersonal | Both genders | Yes (all six items) | (Mandal et al. 2017) |
| Sexual communication - Pande | 1 | Summative | Reliability and validity |  | Other | Individual, Interpersonal | Women/Girls | Yes (all three items) | (Pande et al. 2011) |
| Sexual intercourse and condom use norms - Agha | 2 | Summative | Reliability only (n=2) |  | Social Norms | Individual, Interpersonal | Women/Girls | Yes (all seven items) | (Agha et al. 2020; Agha et al. 2021) |
| Sexual Relationship Power Scale | 1 | Summative | Reliability and validity |  | Other | Interpersonal | Women/Girls; Marginalized population | Yes (four items) | (Parrado et al. 2005) |
| Sexual Self-Efficacy Scale - Bui | 1 | Summative | Reliability and validity | Can |  | Individual | Women/Girls | Yes (all four items) | (Bui et al. 2010) |
| SRH and HH decision making - Jain | 1 | Summative | Reliability and validity | Act |  | Individual | Women/Girls | None | (Jain et al. 2019) |
| Survey-based Women's Empowerment (SWPER) Index | 1 | Summative | Not reported | Act |  | Individual | Women/Girls | Yes (one item) | (Drioui and Bakass 2022) |
| Transactional sexual relations - Fielding-Miler | 1 | Summative | Not reported | Act |  | Individual | Women/Girls; Marginalized population | Yes | (Fielding-Miller et al. 2017) |
| WE-MEASR | 1 | Summative | Reliability and validity | Can, Act |  | Individual, Community | Women/Girls | Yes (two items) | (Boydell et al. 2020) |
| Women’s and Girl’s Empowerment–SRH Index | 1 | Summative | Reliability and validity | Can, Act |  | Individual, Interpersonal | Women/Girls | Yes (all 21 items) | (Moreau et al. 2020) |
| Women's Autonomy scale - Bhandari | 1 | Summative | Reliability and validity | Act | Autonomy | Individual | Women/Girls | Yes (one item) | (Bhandari et al. 2014) |
| Women's empowerment scale - Dasgupta | 1 | Summative | Reliability and validity | Act |  | Individual | Women/Girls | Yes (three items) | (Dasgupta et al. 2016) |
| Work Self-efficacy | 1 | Summative | Not reported | Can |  | Individual | Both genders; Marginalized population | None | (Wagner et al. 2014) |

Note: FP – family planning

**Demographic and Health Survey measures**

**TABLE T6 List of Demographic and Health Survey measures**

| **Journal Article/Resource** | **Type of Measure** | **Measure Construction** | **Psychometric evaluation (if summative)** |
| --- | --- | --- | --- |
| **Can** |  |  |  |
| (Dadras et al. 2022) | Ability to reject sexual relations ore request condom use | Summative | Reliability and validity |
| (Forty et al. 2022) | Ability to reject sexual relations ore request condom use | Summative | Reliability and validity |
| (International Institute for Population 2019) | Fertility Preferences | Categorical | n/a |
|  |  |  |  |
| **Act** |  |  |  |
| (Abekah-Nkrumah 2013) | Decision Making – household, health care, visits to family | Summative | Validity |
| (Adokiya et al. 2021) | Decision Making – household, health care, visits to family | Summative | Reliability |
| (Al Riyami et al. 2004) | Decision Making – family planning, fertility, household, health care, visits to family | Categorical | n/a |
| (Annan et al. 2019) | Decision Making – household | Categorical | n/a |
| (Atteraya et al. 2014) | Decision Making – household, health care, visits to family | Summative | Validity |
| (Blackstone 2017) | Decision Making – household, health care, visits to family | Summative | None |
| (Dadras et al. 2022) | Decision Making – household, health care, visits to family | Summative | Reliability and validity |
| (Dodsworth and Parry 2021) | Decision making – sexual relations, health care | Summative | Reliability and validity |
| (Feyisetan and Oyediran 2020) | Decision Making – household, health care, visits to family | Summative | None |
| (Forty et al. 2022) | Decision Making – household, health care, visits to family | Summative | Reliability and validity |
| (Gebre and Edossa 2020) | Decision Making – family planning | Categorical | n/a |
| (Haque et al. 2021) | Decision Making – family planning, household, health care, visits to family | Summative | Validity |
| (Hossain 2015) | Decision Making – family planning, household, health care, visits to family | Categorical | n/a |
| (International Institute for Population 2019) | Decision Making – family planning, household | Categorical | n/a |
| (Juan et al. 2020) | Decision Making – family planning | Categorical | n/a |
| (León 2013) | Decision Making – household, visits to family | Summative | Reliability and validity |
| (Murugan and Pandey 2019) | Decision Making – household, health care, visits to family | Categorical | n/a |
| (Nazarbegian et al. 2022) | Decision Making – family planning; Value of opinion in decision | Categorical | n/a |
| (Packer et al. 2020) | Decision Making – household, health care | Summative | Reliability |
| (Raisul et al. 2020) | Decision Making – family planning, household, health care, visits to family | Summative | None |
| (Samari 2015) | Decision Making – household, health care | Summative | Reliability and validity |
| (Some et al. 2021) | Decision Making – household, health care | Summative | Reliability |
| (Tuz-Zahura et al. 2022) | Decision Making – household, health care, visits to family | Summative | Reliability |
| (Upadhyay and Karasek 2012) | Decision Making – household, health care, visits to family | Summative | None |
| (Whidden et al. 2021) | Decision Making – household, health care, visits to family; Justification of wife beating | Categorical | n/a |
| (Yaya et al. 2018) | Decision Making – household, health care, visits to family | Summative | Validity |
| (Sedlander et al. 2023) | Decision Making – household, health care | Summative | None |

Note: None of the DHS measures included backlash to agentive action (“resist”)

**TABLE T7 Characteristics of unique measures of agency**

|  | **Can** | | **Act** | | **Resist** | | **Total** |
| --- | --- | --- | --- | --- | --- | --- | --- |
|  | **n** | **%** | **n** | **%** | **n** | **%** | **N** |
| **Age Groups ^a^** |  |  |  |  |  |  |  |
| 10-17 years | 11 | 44.0 | 19 | 76.0 | 1 | 4.0 | 25 |
| 18-29 years | 23 | 57.5 | 23 | 57.5 | 2 | 5.0 | 40 |
| 30-49 years | 17 | 58.6 | 18 | 62.1 | 1 | 3.4 | 29 |
| 50-64 years | 5 | 71.4 | 3 | 42.9 | 0 | 0.0 | 7 |
| 65+ years | 1 | 100.0 | 1 | 100.0 | 0 | 0.0 | 1 |
| **Target Population ^a^** |  |  |  |  |  |  |  |
| Women/Girls | 13 | 52.0 | 17 | 68.0 | 2 | 8.0 | 25 |
| Men/Boys | 2 | 66.7 | 1 | 33.3 | 0 | 0.0 | 3 |
| Women/Girls and Men/Boys | 8 | 66.7 | 5 | 41.7 | 0 | 0.0 | 12 |
| **Region ^a^** |  |  |  |  |  |  |  |
| Sub-Saharan Africa | 18 | 69.2 | 14 | 53.8 | 1 | 3.8 | 26 |
| East Asia & Pacific | 2 | 66.7 | 1 | 33.3 | 0 | 0.0 | 3 |
| Europe & Central Asia | 1 | 33.3 | 2 | 66.7 | 0 | 0.0 | 3 |
| Latin America & the Caribbean | 3 | 60.0 | 2 | 40.0 | 1 | 20.0 | 5 |
| Middle East & North Africa | 3 | 50.0 | 3 | 50.0 | 0 | 0.0 | 6 |
| South Asia | 2 | 20.0 | 9 | 90.0 | 0 | 0.0 | 10 |
| **Context ^a^** |  |  |  |  |  |  |  |
| Rural | 10 | 41.7 | 18 | 75.0 | 1 | 4.2 | 24 |
| Urban | 9 | 45.0 | 15 | 75.0 | 1 | 5.0 | 20 |
| Peri-urban | 7 | 77.8 | 5 | 55.6 | 0 | 0.0 | 9 |
| **Marginalized Population** | 4 | 66.7 | 2 | 33.3 | 0 | 0.0 | 6 |
| **N** | 23 |  | 23 |  | 2 |  | 40 |

Note: a - Categories listed are not mutually exclusive and the row percentages do not add up to 100%.

**References**

Abekah-Nkrumah, Gordon. 2013. "Women's Empowerment and Household Health in Sub-Saharan Africa: Examining the Importance of Social Norms." The University of Manchester. <https://research.manchester.ac.uk/en/studentTheses/womens-empowerment-and-household-health-in-sub-saharan-africa-exa>.

Adokiya, Martin Nyaaba, Michael Boah, and Timothy Adampah. 2021. "Women’s Autonomy and Modern Contraceptive Use in Ghana: A Secondary Analysis of Data from the 2014 Ghana Demographic and Health Survey." *The European Journal of Contraception and Reproductive Health Care* 26 (5):383-89. <https://doi.org/10.1080/13625187.2021.1910234>.

Agha, Sohail, Brooks Morgan, Helena Archer, Shadae Paul, Joseph B. Babigumira, and Brandon L. Guthrie. 2021. "Understanding How Social Norms Affect Modern Contraceptive Use." *BMC Public Health* 21 (1):1061. <https://doi.org/10.1186/s12889-021-11110-2>.

Agha, Sohail, Brooks Morgan, Helena Archer, Shadae Paul, Joseph Babigumira, and Brandon Guthrie. 2020. "Understanding the Mechanism through Which Unfavorable Social Norms Affect Contraceptive Use." *BMC Public Health PREPRINT (Version 1)* <https://doi.org/10.21203/rs.3.rs-116933/v1>

Al Riyami, Asya, Mustafa Afifi, and Ruth M. Mabry. 2004. "Women's Autonomy, Education and Employment in Oman and Their Influence on Contraceptive Use." *Reproductive Health Matters* 12 (23):144-54. <https://www.jstor.org/stable/3775984>

Alemayehu, Mussie, Araya Abrha Medhanyie, Elizabeth Reed, and Afework Mulugeta Bezabih. 2020. "Validation of Family Planning Tool in the Pastoralist Community." *Reproductive Health* 17 (1):123. <https://doi.org/10.1186/s12978-020-00976-x>.

Annan, Jeannie Ruth, Aletheia Amalia Donald, Markus P. Goldstein, Paula Lorena Gonzalez Martinez, and Gayatri B. Koolwal. 2019. "Taking Power: Women's Empowerment and Household Well-Being in Sub-Saharan Africa." The World Bank. <https://ssrn.com/abstract=3485917>.

Arias, María Luisa Flores, Jane Dimmitt Champion, and Norma Elva Sáenz Soto. 2017. "Adaptation of the Contraceptive Self-Efficacy Scale for Heterosexual Mexican Men and Women of Reproductive Age." *Applied Nursing Research* 36:95-99. <https://doi.org/10.1016/j.apnr.2017.06.003>.

Asante, K. O., and P. N. Doku. 2010. "Cultural Adaptation of the Condom Use Self Efficacy Scale (Cuses) in Ghana." *BMC Public Health* 10:227. <https://doi.org/10.1186/1471-2458-10-227>.

Atteraya, Madhu Sudhan, Heejin Kimm, and In Han Song. 2014. "Women's Autonomy in Negotiating Safer Sex to Prevent Hiv: Findings from the 2011 Nepal Demographic and Health Survey." *AIDS Education and Prevention* 26 (1):1-12. <https://doi.org/10.1521/aeap.2014.26.1.1>.

Bhandari, T. R., G. Dangal, P. S. Sarma, and V. R. Kutty. 2014. "Construction and Validation of a Women's Autonomy Measurement Scale with Reference to Utilization of Maternal Health Care Services in Nepal." *JNMA J Nepal Med Assoc* 52 (195):925-34.

Blackstone, Sarah R. 2017. "Women's Empowerment, Household Status and Contraception Use in Ghana." *JOURNAL OF BIOSOCIAL SCIENCE* 49 (4):423-34. <https://doi.org/10.1017/S0021932016000377>.

Boydell, Victoria, Petrus S. Steyn, Joanna Paula Cordero *et al.* 2020. "Adaptation and Validation of Social Accountability Measures in the Context of Contraceptive Services in Ghana and Tanzania." *International Journal for Equity in Health* 19 (1):183. <https://doi.org/10.1186/s12939-020-01286-1>.

Bui, T. C., P. M. Diamond, C. Markham, M. W. Ross, T. A. Nguyen-Le, and L. H. Tran. 2010. "Gender Relations and Sexual Communication among Female Students in the Mekong River Delta of Vietnam." *Cult Health Sex* 12 (6):591-601. <https://doi.org/10.1080/13691050902968769>.

Corroon, M., I. S. Speizer, J. C. Fotso *et al.* 2014. "The Role of Gender Empowerment on Reproductive Health Outcomes in Urban Nigeria." *Matern Child Health J* 18 (1):307-15. <https://doi.org/10.1007/s10995-013-1266-1>.

Dadras, O., M. Dadras, L. Jafari, T. Nakayama, and F. Dadras. 2022. "Women Empowerment and Access to Maternity and Reproductive Healthcare in Pakistan: Cross-Validation of a Survey-Based Index in Afghanistan (Swei-a)." *BMC WOMENS HEALTH* 22 (1):453. <https://doi.org/10.1186/s12905-022-02031-2>.

Dasgupta, Aparajita, Kajari Bandyopadhyay, Lina Bandyopadhyay, Bobby Paul, and Sitikantha Banerjee. 2016. "Does Women Empowerment Predict Contraceptive Use? A Study in a Rural Area of Hooghly District, West Bengal." *Indian Journal of Community Health* 28 (3):228-35.

Dodsworth, Emma, and Luke Parry. 2021. "Reproductive Health Capability: Towards a Tool to Measure Inequity in Reproductive Health. A Case Study of Colombia." Lancaster University (United Kingdom). <https://doi.org/10.17635/lancaster/thesis/1493>.

Drioui, Chaimae, and Fatima Bakass. 2022. "Fertility Preferences and Outcomes in Morocco: Does Women's Empowerment Matter in Actual-Ideal Gap?" *Population Review* 61 (1):1. <https://doi.org/10.1353/prv.2022.0000>.

Feldman, B. S., A. M. Zaslavsky, M. Ezzati, K. E. Peterson, and M. Mitchell. 2009. "Contraceptive Use, Birth Spacing, and Autonomy: An Analysis of the Oportunidades Program in Rural Mexico." *Stud Fam Plann* 40 (1):51-62. <https://doi.org/10.1111/j.1728-4465.2009.00186.x>.

Feyisetan, Bamikale, and Kola A. Oyediran. 2020. "Can Married or Cohabiting Women Negotiate Protective Sex? Findings from Demographic and Health Surveys of Two West African Countries." *JOURNAL OF BIOSOCIAL SCIENCE* 52 (6):785-808. <https://doi.org/10.1017/S0021932019000798>.

Fielding-Miller, Rebecca, Kristin L. Dunkle, Craig Hadley, Hannah L. Cooper, and Michael Windle. 2017. "Agency as a Mediator in the Pathway from Transactional Sex to Hiv among Pregnant Women in Swaziland: A Multigroup Path Analysis." *J Int AIDS Soc* 20 (1):21554. <https://doi.org/10.7448/ias.20.1.21554>.

Forty, James, Navaneetham Kannan, and Gobopamang Letamo. 2022. "Determinants of Fertility in Malawi: Does Women Autonomy Dimension Matter?" *BMC Women's Health* 22:1-16. <https://doi.org/10.1186/s12905-022-01926-4>.

Gebre, Mamo Nigatu, and Zerihun Kura Edossa. 2020. "Modern Contraceptive Utilization and Associated Factors among Reproductive-Age Women in Ethiopia: Evidence from 2016 Ethiopia Demographic and Health Survey." *BMC Women's Health* 20 (1):1-14. <https://doi.org/10.1186/s12905-020-00923-9>.

Giménez-García, Cristina, Estefanía Ruiz-Palomino, María Dolores Gil-Llario, Rafael Ballester-Arnal, and Claudia Castañeiras. 2018. "Why Do Young Hispanic Women Take Sexual Risks? Psychological and Cultural Factors for Hiv Prevention." *JANAC: Journal of the Association of Nurses in AIDS Care* 29 (5):762-69. <https://doi.org/10.1016/j.jana.2018.05.006>.

Ha, Bui Thi Thu, Rohan Jayasuriya, and Neville Owen. 2003. "Male Involvement in Family Planning in Rural Vietnam: An Application of the Transtheoretical Model." *Health education research* 18 2:171-80.

Hamid, Saima, Rob Stephenson, and Birgitta Rubenson. 2011. "Marriage Decision Making, Spousal Communication, and Reproductive Health among Married Youth in Pakistan." *Global Health Action* 4 (1):5079. <https://doi.org/10.3402/gha.v4i0.5079>.

Haque, Rezwanul, Khorshed Alam, Syed Mahbubur Rahman, Keramat Syed Afroz, and Mohammed Khaled Al-Hanawi. 2021. "Women’s Empowerment and Fertility Decision-Making in 53 Low and Middle Resource Countries: A Pooled Analysis of Demographic and Health Surveys." *BMJ Open* 11 (6)<https://doi.org/10.1136/bmjopen-2020-045952>.

Hinson, Laura, Jeffrey Edmeades, Lydia Murithi, and Mahesh Puri. 2019. "Developing and Testing Measures of Reproductive Decision-Making Agency in Nepal." *SSM Popul Health* 9:100473. <https://doi.org/10.1016/j.ssmph.2019.100473>.

Hossain, Belayet. 2015. "Women Empowerment and Infant Mortality in Bangladesh." *Applied Economics* 47 (51):5534-47. <https://doi.org/10.1080/00036846.2015.1051657>.

Hossain, M. K., and M. Kabir. 2001. "Purdah, Mobility and Women's Empowerment and Reproductive Behaviour in Rural Bangladesh." *Social Change* 31 (3):84-102. <https://doi.org/10.1177/004908570103100307>.

International Institute for Population, Sciences. 2019. "National Family Health Survey, India 2019-20: Women's Questionnaire." <http://rchiips.org/NFHS/about.shtml>

Jain, A., H. Ismail, E. Tobey, and A. Erulkar. 2019. "Stigma as a Barrier to Family Planning Use among Married Youth in Ethiopia." *J Biosoc Sci* 51 (4):505-19. <https://doi.org/10.1017/S0021932018000305>.

Juan, C., C. Allen, and K. L. D. MacQuarrie. 2020. "Associations between Women’s Current Contraceptive Method Decision Making and Their Reproductive Calendar Histories in Burundi." ICF.

Kahsay, Znabu H., Dessie Tegegne, Ebrahim Mohammed, and Getachew Kiros. 2018. "Application of Individual Behavioral Models to Predict Willingness to Use Modern Contraceptives among Pastoralist Women in Afar Region, Northern Ethiopia." *PLOS ONE* 13 (5):e0197366. <https://doi.org/10.1371/journal.pone.0197366>.

Kamiya, Yusuke. 2010. *Endogenous Women's Autonomy and the Use of Reproductive Health Services: Empirical Evidence from Tajikistan*. Federal Reserve Bank of St Louis.

Kiani, Zahra, Masoumeh Simbar, Mahrokh Dolatian, and Farid Zayeri. 2020. "Structural Equation Modeling of Psychosocial Determinants of Health for the Empowerment of Iranian Women in Reproductive Decision Making." *BMC Women's Health* 20 (1):1-9. <https://doi.org/10.1186/s12905-020-0893-0>.

Kridli, S. A., and K. Libbus. 2002. "Establishing Reliability and Validity of an Instrument Measuring Jordanian Muslim Women's Contraceptive Beliefs." *Health Care Women Int* 23 (8):870-81. <https://doi.org/10.1080/07399330290112371>.

León, F. R. 2013. "Predicting Contraceptive Use from an Egalitarian Model of Women's Overall Household Power Vis-À-Vis Conventional Power Models and Third Variables." *J Biosoc Sci* 45 (4):497-515. <https://doi.org/10.1017/s0021932012000624>.

Loll, Dana, Paul J. Fleming, Abubakar Manu *et al.* 2019. "Reproductive Autonomy and Modern Contraceptive Use at Last Sex among Young Women in Ghana." *International Perspectives on Sexual and Reproductive Health* 45:1-12. <https://doi.org/10.1363/45e7419>.

Mandal, Mahua, and Lisa M. Albert. 2020. "Reproductive Empowerment Scale: Psychometric Validation in Nigeria." MEASURE Evaluation, University of North Carolina. <https://www.measureevaluation.org/resources/publications/tr-20-393>.

Mandal, Mahua, Arundati Muralidharan, and Sara Pappa. 2017. "A Review of Measures of Women’s Empowerment and Related Gender Constructs in Family Planning and Maternal Health Program Evaluations in Low- and Middle-Income Countries." *BMC Pregnancy and Childbirth* 17 (2):342. <https://doi.org/10.1186/s12884-017-1500-8>.

McCarthy, Ona L., Hanadi Zghayyer, Amina Stavridis *et al.* 2019. "A Randomized Controlled Trial of an Intervention Delivered by Mobile Phone Text Message to Increase the Acceptability of Effective Contraception among Young Women in Palestine." *Trials* 20 (1):228. <https://doi.org/10.1186/s13063-019-3297-4>.

Moreau, Caroline, Caroline Karp, Shannon N. Wood *et al.* 2020. "Reconceptualizing Women's and Girls' Empowerment: A Cross-Cultural Index for Measuring Progress toward Improved Sexual and Reproductive Health." *Int Perspect Sex Reprod Health* 46:187-98. <https://doi.org/10.1363/46e9920>.

Murugan, Vithya, and Shanta Pandey. 2019. "Correlates of Female Sterilization in Bihar: Does Women’s Empowerment Matter?" *GLOBAL SOCIAL WELFARE* 6 (2):79-85. <https://doi.org/10.1007/s40609-018-0116-x>.

Nazarbegian, M., S. Averbach, N. E. Johns *et al.* 2022. "Associations between Contraceptive Decision-Making and Marital Contraceptive Communication and Use in Rural Maharashtra, India." *Stud Fam Plann* 53 (4):617-37. <https://doi.org/10.1111/sifp.12214>.

Newmann, Sara J., Jennifer Monroe Zakaras, Shari L. Dworkin *et al.* 2021. "Measuring Men’s Gender Norm Beliefs Related to Contraception: Development of the Masculine Norms and Family Planning Acceptance Scale." *ARCHIVES OF SEXUAL BEHAVIOR* <https://doi.org/10.1007/s10508-021-01941-w>.

Okigbo, Chinelo C., Ilene S. Speizer, Marisa E. Domino, Sian L. Curtis, Carolyn T. Halpern, and Jean C. Fotso. 2018. "Gender Norms and Modern Contraceptive Use in Urban Nigeria: A Multilevel Longitudinal Study." *BMC Women's Health* 18 (1):178. <https://doi.org/10.1186/s12905-018-0664-3>.

Packer, Catherine A., Sayed Haroon Rastagar, Mario Chen *et al.* 2020. "Factors Associated with Reported Modern Contraceptive Use among Married Men in Afghanistan." *REPRODUCTIVE HEALTH* 17 (1):64. <https://doi.org/10.1186/s12978-020-0908-1>.

Paek, Hye-Jin, Byoungkwan Lee, Charles T. Salmon, and Kim Witte. 2008. "The Contextual Effects of Gender Norms, Communication, and Social Capital on Family Planning Behaviors in Uganda: A Multilevel Approach." *Health Education & Behavior* 35 (4):461-77. <https://doi.org/10.1177/1090198106296769>.

Pande, R. P., T. Y. Falle, S. Rathod *et al.* 2011. "'If Your Husband Calls, You Have to Go': Understanding Sexual Agency among Young Married Women in Urban South India." *Sexual Health (14485028)* 8 (1):102-09. <https://doi.org/10.1071/SH10025>.

Parrado, E. A., C. A. Flippen, and C. McQuiston. 2005. "Migration and Relationship Power among Mexican Women." *Demography* 42 (2):347-72. <https://doi.org/10.1353/dem.2005.0016>.

Raisul, Akram, Sarker Abdur Razzaque, Nurnabi Sheikh, Ali Nausad, M. G. N. Mozumder, and Marufa Sultana. 2020. "Factors Associated with Unmet Fertility Desire and Perceptions of Ideal Family Size among Women in Bangladesh: Insights from a Nationwide Demographic and Health Survey." *PLOS ONE* 15 (5)<https://doi.org/10.1371/journal.pone.0233634>.

Richardson, Emma. 2016. "'Uno Tiene Que Cuidar Tambien De Si Mismo': Guatemalan Family Planning Decisions in the Context of Social Cognitive Theory and a Political Economy Approach." 76 <https://hdl.handle.net/1807/68387>

Samanta, Tannistha. 2020. "Women’s Empowerment as Self-Compassion?: Empirical Observations from India." *PLoS One* 15 (5):e0232526. <https://doi.org/10.1371/journal.pone.0232526>.

Samari, Goleen. 2015. "Women's Status, Autonomy, and Fertility in Transitional Egypt." 76 UCLA. <https://escholarship.org/uc/item/8cm0p7vf>.

Sedlander, Erica, Minakshi Dahal, Jeffrey Bart Bingenheimer *et al.* 2023. "Adapting and Validating the G-Norm (Gender Norms Scale) in Nepal: An Examination of How Gender Norms Are Associated with Agency and Reproductive Health Outcomes." *Studies in Family Planning* 54 (1):181-200. <https://doi.org/10.1111/sifp.12231>.

Silva, M., K. Edan, and L. Dougherty. 2021. "Monitoring the Quality Assurance Branding Campaign Confiance Totale in Côte D’ivoire." Breakthrough RESEARCH. <https://breakthroughactionandresearch.org/quality-assurance-confiance-totale-cote-divoire/>.

Singh, Kavita, Ilene S. Speizer, E. Ijdi Rashida, and Lisa M. Calhoun. 2021. "The Association of Empowerment Measures with Maternal, Child and Family Planning Outcomes in Plateau State Nigeria by Urban-Rural Residence." *BMC Pregnancy and Childbirth* 21:1-14. <https://doi.org/10.1186/s12884-021-03659-y>.

Some, Sylvain Y. M., Christy Pu, and Song-Lih Huang. 2021. "Empowerment and Use of Modern Contraceptive Methods among Married Women in Burkina Faso: A Multilevel Analysis." *BMC Public Health* 21:1-13. <https://doi.org/10.1186/s12889-021-11541-x>.

Somji, Aleefia, Kate Ramsey, Sean Dryer *et al.* 2022. "“Taking Care of Your Pregnancy”: A Mixed-Methods Study of Group Antenatal Care in Kakamega County, Kenya." *BMC Health Services Research* 22:1-15. <https://doi.org/10.1186/s12913-022-08200-1>.

Sudhinaraset, M., P. A. Afulani, N. Diamond-Smith, G. Golub, and A. Srivastava. 2018. "Development of a Person-Centered Family Planning Scale in India and Kenya." *Stud Fam Plann* 49 (3):237-58. <https://doi.org/10.1111/sifp.12069>.

Sudhinaraset, May, Amanda Landrian, Patience A. Afulani, Beth Phillips, Nadia Diamond-Smith, and Sun Cotter. 2020. "Development and Validation of a Person-Centered Abortion Scale: The Experiences of Care in Private Facilities in Kenya." *BMC Women's Health* 20 (1):208. <https://doi.org/10.1186/s12905-020-01071-w>.

Tuz-Zahura, Fatima, Kanchan Kumar Sen, Nilima Shahnaz, and Wasimul Bari. 2022. "Can Women’s 3e Index Impede Short Birth Interval? Evidence from Bangladesh Demographic and Health Survey, 2017–18." *PLOS ONE* 17 (1)<https://doi.org/10.1371/journal.pone.0263003>.

Upadhyay, Ushma D., and Deborah Karasek. 2012. "Women's Empowerment and Ideal Family Size: An Examination of Dhs Empowerment Measures in Sub-Saharan Africa." *International Perspectives on Sexual and Reproductive Health* 38 (2):78-89. <https://doi.org/10.1363/3807812>.

Wagner, Glenn J., Victoria K. Ngo, Noeline Nakasujja, Dickens Akena, Frances Aunon, and Seggane Musisi. 2014. "Impact of Antidepressant Therapy on Cognitive Aspects of Work, Condom Use, and Psychosocial Well-Being among Hiv Clients in Uganda." *International Journal of Psychiatry in Medicine* 48 (3):155-66. <https://doi.org/10.2190/PM.48.3.a>.

Waszak, Cynthia, Lawrence J. Severy, Laila Kafafi, and Isis Badawi. 2001. "Fertility Behavior and Psychological Stress: The Mediating Influence of Gender Norm Beliefs among Egyptian Women." *Psychology of Women Quarterly* 25 (3):197-208. <https://doi.org/10.1111/1471-6402.00021>.

Wegs, C., A. A. Creanga, C. Galavotti, and E. Wamalwa. 2016. "Community Dialogue to Shift Social Norms and Enable Family Planning: An Evaluation of the Family Planning Results Initiative in Kenya." *PLoS One* 11 (4):e0153907. <https://doi.org/10.1371/journal.pone.0153907>.

Whidden, Caroline, Youssouf Keita, Emily Treleaven *et al.* 2021. "Women’s Empowerment, Intrahousehold Influences, and Health System Design on Modern Contraceptive Use in Rural Mali: A Multilevel Analysis of Cross-Sectional Survey Data." *REPRODUCTIVE HEALTH* 18:1-16. <https://doi.org/10.1186/s12978-020-01061-z>.

Whiting-Collins, L., L. Grenier, P. J. Winch, A. Tsui, and P. K. Donohue. 2020. "Measuring Contraceptive Self-Efficacy in Sub-Saharan Africa: Development and Validation of the Csessa Scale in Kenya and Nigeria." *Contracept X* 2:100041. <https://doi.org/10.1016/j.conx.2020.100041>.

Whiting-Collins, Lillian Joyce. 2021. "Postpartum Family Planning among Women Attending Group-Based Antenatal and Postnatal Care in Kenya and Nigeria: A Cluster Randomized Control Trial." 82 Johns Hopkins University. <http://jhir.library.jhu.edu/handle/1774.2/62596>.

Yang, Xueyan, Shuzhuo Li, Zheng Wu, and Christoph M. Schimmele. 2009. "Developing Scales for Measuring Gender Behaviors in Reproductive Health in Rural China." *Biodemography Soc Biol* 55 (1):82-92. <https://doi.org/10.1080/19485560903054721>.

Yaya, S., O. A. Uthman, M. Ekholuenetale, and G. Bishwajit. 2018. "Women Empowerment as an Enabling Factor of Contraceptive Use in Sub-Saharan Africa: A Multilevel Analysis of Cross-Sectional Surveys of 32 Countries." *Reprod Health* 15 (1):214. <https://doi.org/10.1186/s12978-018-0658-5>.
